# Biaxial Cyclic Loading Test for Bauschinger Effect Characterization of Q890 High-Strength Steel

**DOI:** 10.3390/ma19143025

**Published:** 2026-07-14

**Authors:** Lin Zhu, Shuo Wang, Yanli Lin, Yuetong Li, Bingyan Jing, Yibo Su, Leheng Huang, Chunyu Ou, Yingguang Zhao, Xiangyue Sun, Zhubin He

**Affiliations:** 1 State Key Laboratory of High-Performance Precision Manufacturing, Dalian University of Technology, Dalian 116024, China; lin_zhulin123@163.com (L.Z.); wshuo98@mail.dlut.edu.cn (S.W.); liyuetong2023@163.com (Y.L.); nefujby@163.com (B.J.); suyibo2021@163.com (Y.S.); hlh26@mail.dlut.edu.cn (L.H.); ouchunyu1415@163.com (C.O.);; 2 School of Mechanical Engineering, Dalian University of Technology, Dalian 116024, China

**Keywords:** Q890 high-strength steel, cyclic four-point bending, Bauschinger effect, in-plane biaxial stress state, springback behavior

## Abstract

Large-scale thick curved components made of high-strength steel are critical to deep-sea pressure hulls and large structural components of engineering machinery. During forming, these components experience reverse loading upon unloading, and the pronounced Bauschinger effect of high-strength steel significantly compromises springback prediction accuracy, leading to costly die iterations. Existing cyclic tension–compression and shear tests are limited to uniaxial stress states and fail to capture the mechanical behavior under in-plane biaxial cyclic loading. Herein, a cyclic four-point bending method is proposed to characterize the Bauschinger effect of Q890 steel under biaxial cyclic loading. By tailoring the width-to-thickness ratio of the specimens, a series of plane stress states with different initial plastic stress ratios were obtained, covering the dominant stress conditions encountered in forming typical large-scale double-curvature thick plates. Full-field strain evolution during cyclic bending was captured in real time via digital image correlation (DIC), enabling systematic acquisition of equivalent stress–strain curves under various biaxial stress ratios over multiple cycles. As the width-to-thickness ratio increases, both forward and reverse yielding progressively degrade: the equivalent yield strength, forward peak flow stress, and reverse yield strength drop from 1098, 1193, and 742 MPa to 934, 1065, and 685 MPa, respectively. Accordingly, the Bauschinger ratio B, Bauschinger hardening parameter BHP, and Bauschinger energy parameter BEP decrease from 0.479, 0.789, and 4.747 to 0.363, 0.655, and 2.900, respectively, revealing a strong stress-ratio dependence of the Bauschinger effect. Notably, the springback ratio also shows clear dependence on the biaxial stress ratio, loading direction, and cyclic history, indicating that in-plane biaxial stress-state effects should be considered when characterizing the Bauschinger effect and springback behavior of Q890 high-strength steel.

## 1. Introduction

For high-strength steel sheet forming, springback is no longer merely a geometric recovery after unloading, but an accuracy issue governed by the combined effects of material strength, elastic unloading, residual stress, and loading path. With the increasing use of high-strength steel in construction machinery, bridge structures, marine equipment, and high-end load-bearing components, its high yield strength and large elastic strain energy make springback after forming more pronounced, leading to profile deviation, curvature error, and reduced assembly consistency [1,2,3].

During multi-pass and reverse bending, the material undergoes successive loading, unloading, and path reversal, where residual stress, nonlinear unloading–reloading, and the Bauschinger effect jointly alter the springback response [4]. Therefore, springback prediction based primarily on material parameters obtained from a single loading path may lead to limited accuracy in describing the actual deformation recovery behavior of high-strength steel during complex bending forming processes [5,6,7]. The Bauschinger effect is a key factor in interpreting the reverse-loading behavior of high-strength steel and the associated errors in springback prediction. After pre-deformation, the evolution of internal back stress and dislocation structures reduces the yield stress upon reverse loading, leading to pronounced loading-path dependence [8,9,10]. To address this issue, previous studies have mainly investigated three types of loading paths: tension–compression, shear, and bending. Cyclic tension–compression tests can directly capture the yield transition between forward and reverse loading and are therefore widely used to calibrate kinematic hardening models and the Yoshida–Uemori model [11,12]. Previous studies have shown that neglecting cyclic plasticity features, such as reverse yield reduction and transient hardening, can lead to significant deviations in springback angle prediction [13,14]. However, tension–compression tests are generally dominated by a uniaxial stress state, which limits the applicability of the obtained parameters to complex bending forming processes involving multiaxial stress interactions.

To avoid compressive buckling commonly encountered in cyclic tension–compression tests of thin sheets, shear/reverse-shear tests have also been widely used to characterize the Bauschinger effect and identify cyclic hardening parameters. This method enables stable reverse-loading curves to be obtained over a relatively large plastic strain range and provides a basis for parameter identification of mixed hardening models, such as the Chaboche model [15,16,17,18]. Previous studies have modified springback simulation parameters based on shear loading paths, thereby improving the prediction accuracy for high-strength steel to some extent. However, shear tests mainly characterize the cyclic response under an approximately pure simple-shear state, whose stress path is not fully consistent with the tension–compression transition, through-thickness stress gradient, and lateral constraint experienced by the surface layer during actual bending forming [19]. Therefore, springback prediction based solely on shear-calibrated parameters may still underestimate or neglect the influence of stress-state evolution during bending on reverse yielding and springback behavior.

Classical methods [20], such as the cruciform tension–compression test and the hydraulic bulging test, have been widely employed to evaluate the mechanical behavior of sheet metals. However, although the hydraulic bulging test is a standard method for evaluating biaxial tension, it is physically incapable of performing the reverse compressive loading required to capture the Bauschinger effect. Similarly, while cruciform specimens can theoretically achieve in-plane reverse loading, applying compressive strain to macroscopic heavy plates frequently triggers buckling. To address the need for reverse loading, bending-reverse-bending (BRB) tests have been widely utilized to characterize the Bauschinger effect in sheet materials, in which the inner and outer layers sequentially experience compression-tension and tension–compression deformations. For instance, Yoshida et al. [21,22] designed a cyclic bending test apparatus for thin sheet metals and proposed an advanced parameter optimization technique based on measured moment-curvature curves. Since this foundational work, determining the cyclic stress–strain response via inverse numerical methods has become highly prevalent [23]. Alternatively, Zang et al. [24] proposed evaluating the Bauschinger effect by subjecting pre-tensioned specimens to subsequent three-point bending. Furthermore, three-point [24] and four-point bending tests [25,26] closely reproduce the actual bending deformation of sheet metals and can be used to analyze reverse yielding, transient hardening, and springback variations after pre-deformation. In particular, four-point bending tests generate a relatively stable pure-bending region between the two loading rollers, facilitating the analysis of the mechanical response in the target region when combined with surface strain measurements. However, most existing bending tests employ narrow specimens or fixed geometries and primarily focus on the effects of bending radius, pre-strain, or loading path on springback. Consequently, insufficient attention has been paid to the differences in in-plane stress states induced by variations in the specimen’s width-to-thickness ratio.

During actual forming, the sheet surface is not always subjected to an ideal uniaxial tension–compression state. Variations in width-to-thickness ratio, lateral constraint, and bending stiffness can generate a transverse stress component $\sigma_{11}$ in addition to the longitudinal bending stress $\sigma_{22}$, resulting in an evolving in-plane biaxial stress state during loading. Consequently, the Bauschinger effect and springback behavior under such biaxial cyclic loading cannot be accurately described by conventional tests that assume a uniaxial or pure-shear stress state.

Recent studies on biaxial loading and stress-state-sensitive constitutive modeling have shown that different stress states can significantly affect anisotropic hardening, reverse yielding, and springback prediction accuracy [27,28]. Zhang and Lou [29] reported that bending can involve a wide range of stress states, from uniaxial stress to near-plane-strain conditions, and that the accuracy of V-bending springback prediction is limited when such differences are not properly captured in the constitutive description [20,30]. This indicates that although tension–compression, shear, and conventional narrow-specimen bending tests can reveal loading-path-dependent plastic behavior, they are still insufficient to fully explain the differences in the biaxial stress state on the target surface induced by variations in the width-to-thickness ratio, as well as their effects on the Bauschinger effect and cyclic bending springback. Therefore, it is necessary to establish the relationships among the width-to-thickness ratio, stress ratio, reverse-loading response, and springback behavior from the perspective of the target-surface stress state.

This study proposes a cyclic four-point bending test method for characterizing the Bauschinger effect under in-plane biaxial cyclic loading. Unlike conventional bending tests that treat the specimen as a narrow beam, the present method deliberately varies the width-to-thickness ratio to achieve a series of stress states with biaxial stress ratios $\alpha_{t}=\sigma_{11}/\sigma_{22}$, covering the dominant stress conditions encountered in forming typical large-scale double-curvature thick plates. Full-field strain evolution during cyclic bending is captured in real time via DIC, enabling the systematic acquisition of equivalent stress–strain curves under various biaxial stress ratios over multiple cycles. By analyzing the forward yield stress, forward flow stress, reverse yield stress, and Bauschinger-related parameters, the effects of the in-plane biaxial stress state on reverse yielding, cyclic stress asymmetry, and springback behavior are clarified.

## 2. Materials and Methods

### 2.1. Experimental Material and Specimen Preparation

Q890 high-strength steel (Henan Xiangwu Steel Co., Ltd., Zhengzhou, China) in the quenched and tempered condition was used in this study. Its main chemical composition is listed in Table 1.

**Figure 1:**
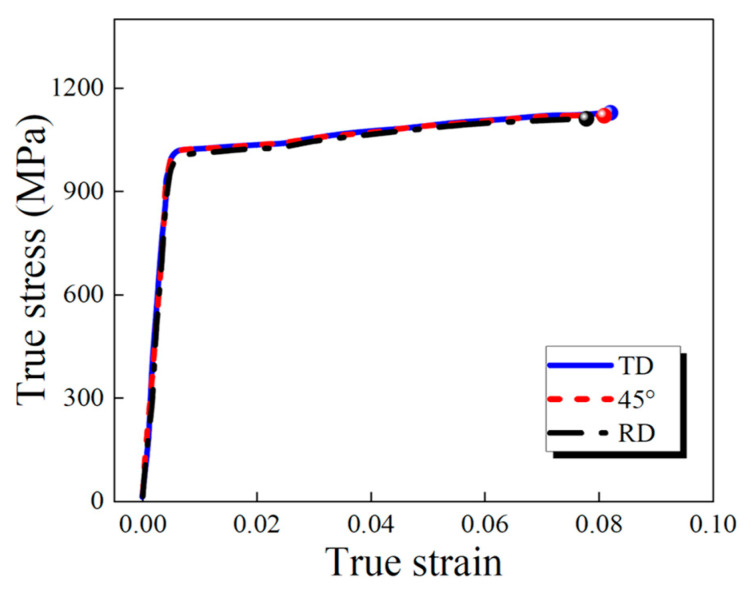
True stress–strain curves of Q890 steel sheet.

Uniaxial tensile specimens were extracted along the rolling direction (RD), diagonal direction (45°), and transverse direction (TD) of the steel plate. Uniaxial tensile tests were performed at room temperature under a loading rate of 1 mm/min. To ensure experimental reproducibility and statistical reliability, at least three duplicate tests were conducted for each orientation, and the representative average engineering responses were converted into true stress–strain curves, as illustrated in Figure 1. Utilizing the plastic strain data from the uniaxial tensile tests, the anisotropy coefficients were evaluated in compliance with the GB/T 5027-2016 standard [31]. Table 2 lists the mechanical properties of the Q890 steel plate. The mechanical properties of the Q890 steel are basically the same in the three directions, and the difference in anisotropy is very small. Therefore, the effect of material anisotropy is not considered in this study.

**Figure 2:**
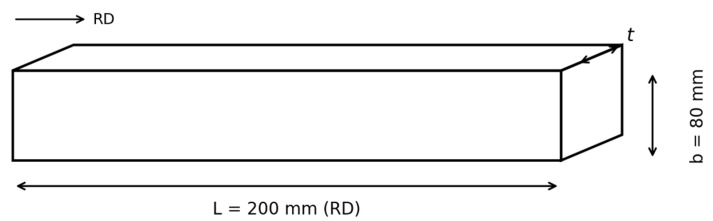
Geometry and dimensions of the rectangular four-point bending specimen.

To ensure consistency between experimental characterization and numerical simulation in terms of geometric boundary conditions, rectangular specimens were used, with a length of L = 200 mm and a width of b = 80 mm. The specimen length direction was aligned with the rolling direction (RD), as shown in Figure 2. The specimens were prepared from quenched and tempered Q890 steel plates by wire cutting. To minimize the influence of machining burrs and localized stress concentrations on local strain measurements, the edges of the bending region were systematically ground and polished using progressively finer abrasive papers. The detailed testing conditions are described in Section 2.3.

### 2.2. Construction of In-Plane Biaxial Stress States and Representative Test Conditions

To characterize the Bauschinger effect under different in-plane stress states, a finite element model of cyclic four-point bending was first established to investigate the effects of the specimen width-to-thickness ratio on $\sigma_{11}$, $\sigma_{22}$ and the biaxial stress ratio $\alpha_{t}$ on the target surface. Unlike conventional uniaxial tension–compression or shear tests, in which the loading path is directly prescribed, the present method constructs representative in-plane biaxial stress states by adjusting the width-to-thickness ratio b/t, thereby changing the lateral constraint, bending stiffness, and transverse stress component on the target surface.

#### 2.2.1. Statistical Analysis of Stress States in Heavy-Plate Forming

**Figure 3:**
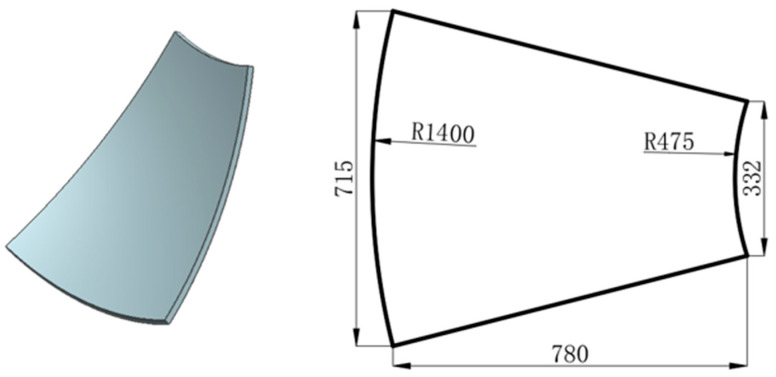
Target Shape and Dimensions of the Initial Blank (Unit: mm).

The geometry of the target component for the Q890 high-strength steel heavy plate is illustrated in Figure 3. Its inner surface is a spherical surface with a curvature radius of 1200 mm. The arc lengths of the large and small ends are 592 mm and 273 mm, respectively, while the lengths of the straight segments on both sides are 640 mm. To enhance forming accuracy, a machining allowance of 50 mm is designated for the curved edges on both sides of the blank, and a machining allowance of 40 mm is allocated for each side of the straight segments. The geometry and dimensions of the initial blank are shown in the figure, with a plate thickness of 16 mm.

**Figure 4:**
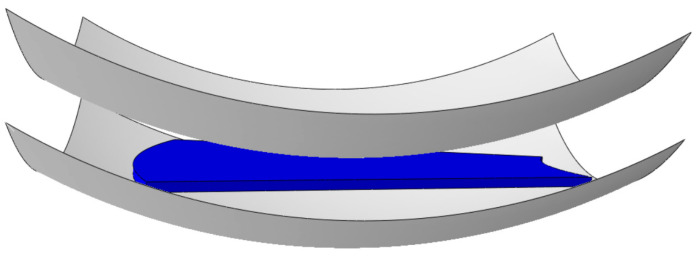
Finite element model for integral press forming of Q890 high-strength steel thick plate. The gray parts represent the die/mold, and the blue parts represent the slab/blank.

To ensure that the finite element simulation model accurately reflects the geometric characteristics of the target component, the upper die utilizes a spherical surface with a curvature radius identical to that of the target component’s inner surface, and the lower die utilizes a spherical surface consistent with the curvature radius of the outer surface. Based on the curvature parameters of the target component’s inner and outer surfaces, the curvature radii of the upper and lower dies are set to 1200 mm and 1216 mm, respectively. Simultaneously, to ensure that the dies adequately cover the effective forming area and to mitigate the interference of edge effects on the computational results, the dimensions of both the upper and lower dies are set to 1000 mm in length and 800 mm in width. Based on these configurations, the ABAQUS finite element simulation model was established, as schematically illustrated in Figure 4.

The finite element (FE) simulation was conducted using a dynamic explicit algorithm. The upper and lower dies were meshed with shell elements and defined as rigid bodies, while the blank was defined as a deformable body. Surface-to-surface contact was established between the dies and the blank. The friction type was set to steel-to-steel contact with a friction coefficient of 0.15, and the mesh size was uniformly set to 8 mm × 8 mm × 8 mm. To ensure the stability of the forming process, the upper die first descended at a velocity of 5 mm/s and stopped at a distance of 2 mm from the upper surface of the blank, after which gravity was applied and maintained for 0.5 s. Subsequently, the lower die moved downward by 102 mm within 2 s to adjust the clearance to 2 mm. The upper die then continued to press downward by 2 mm within 0.5 s to complete the die closure process. After maintaining the pressure for 0.5 s, the dies were opened, and the blank underwent springback deformation.

**Figure 5:**
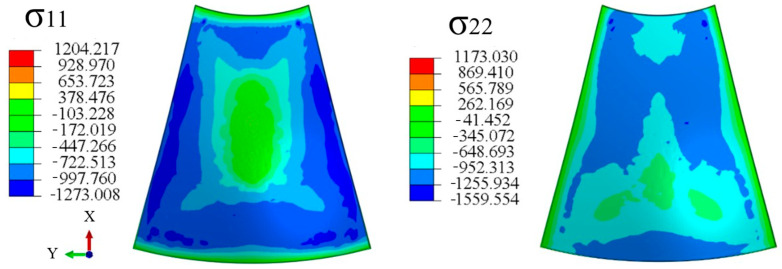
FE simulation of large-scale double-curvature thick-plate forming.

**Figure 6:**
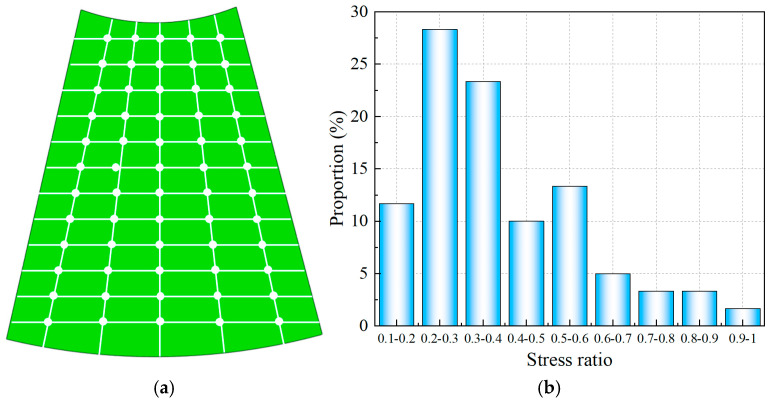
Statistical results of stress ratio. (**a**) Schematic of sampling point locations. (**b**) Statistical stress ratios.

Figure 5 presents the stress distribution diagram of the high-strength steel heavy-plate component during the forward pressing process, which can be utilized to analyze the stress states during forming. Further observation of the in-plane stress contours reveals that although σ_11_ and σ_22_ are generally in the same order of magnitude, their distribution patterns and relative magnitudes exhibit significant differences across various regions. This indicates that while the component macroscopically undergoes the same pressing displacement, the actual combinations of in-plane stresses experienced by material elements at different spatial locations are inconsistent. To quantitatively analyze the stress state distribution characteristics on the inner surface of the large-scale curved component, numerous sampling points were uniformly arranged along the primary deformation zone on the inner surface of the finite element model, as shown in Figure 6a. Considering that the near-zero stress stage at the onset of loading and the stress redistribution during unloading would interfere with the statistical results, the stress data at each sampling point during the initial plastic state phase were selected for statistical analysis. The stress ratios of the sampling points on the inner surface were statistically analyzed, and the results are presented in Figure 6b. During the initial yielding stage, the localized stress ratios across the majority of the plate surface are predominantly concentrated within a range of 0.10 to 0.40.

#### 2.2.2. Calibration of Chaboche Mixed Hardening Parameters Based on Cyclic Shear Tests

**Figure 7:**
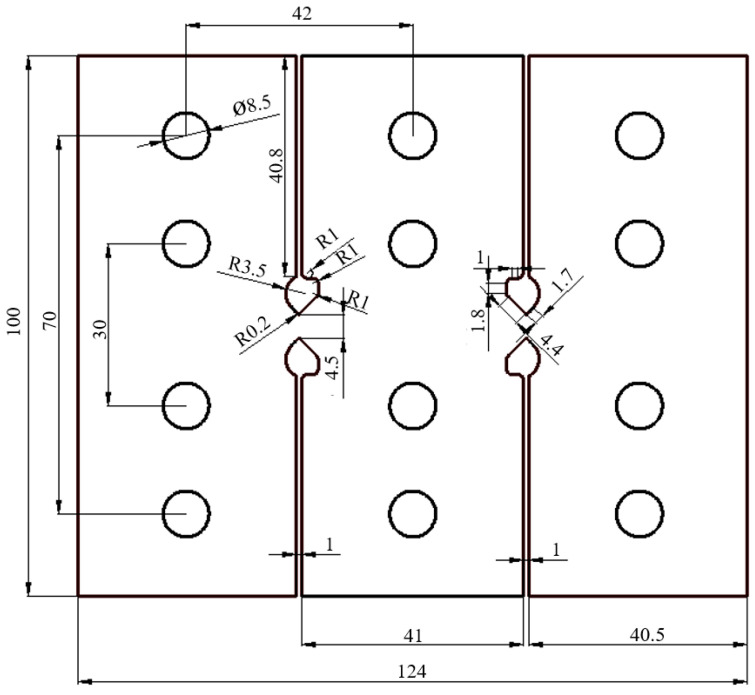
Geometry and dimensions of the shear specimen (Unit: mm).

**Figure 8:**
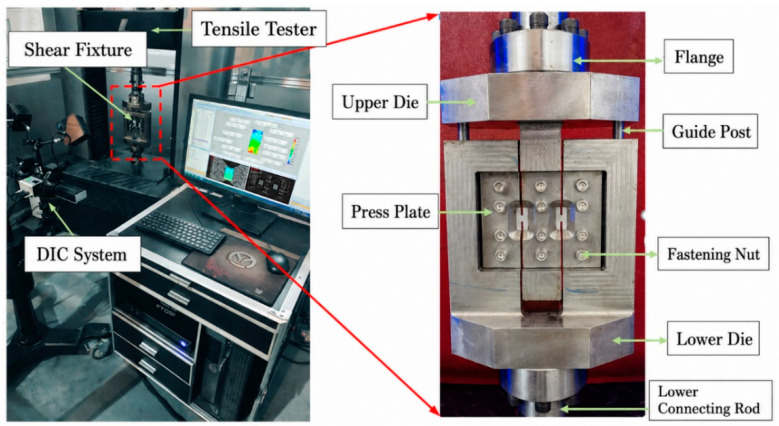
Shear testing system and fixture for sheet metal.

The cyclic elastoplastic behavior of Q890 high-strength steel was described using the Chaboche mixed hardening model, which can effectively capture the Bauschinger effect and permanent softening under reverse loading. To identify the model parameters, cyclic shear tests were further conducted. The shear specimen geometry and the corresponding cyclic shear testing system (self-developed by Dalian University of Technology, Dalian, China) are shown in Figure 7 and Figure 8, respectively.

The Chaboche mixed hardening model parameters were calibrated using the stress–strain data obtained from cyclic shear tests. The model, which consists of isotropic and kinematic hardening components, is expressed as:

(1) $$f=\sqrt{\left(\frac{3}{2}\left(\sigma^{\prime }-\alpha^{\prime }\right):\left(\sigma^{\prime }-\alpha^{\prime }\right)\right)}-\sigma_{y0}-r\left(p\right)$$

where $\sigma^{\prime }$ is the deviatoric stress tensor, $\alpha^{\prime }$ is the kinematic hardening variable, namely the backstress, $\sigma_{y0}$ is the initial yield stress, and r(p) is the isotropic hardening function, expressed as:

(2) $$r\left(p\right)=Q\left(1-e^{-b^{\prime }p}\right)$$

where *Q* and *b′* are material parameters. *Q* can be obtained by interpolation between the peak stress and the initial yield stress, while *b′* controls the rate at which the stress approaches the peak value. p denotes the accumulated plastic strain.

For the kinematic hardening component, the backstress increment in nonlinear kinematic hardening can be expressed as:

(3) $$d\alpha =\frac{2}{3}cd\varepsilon^{p}-\gamma \alpha dp$$

where *c* and *γ* are material parameters, $d\varepsilon^{p}$ is the plastic strain increment, and dp is the equivalent plastic strain increment.

The Chaboche mixed hardening model implemented in ABAQUS allows multiple backstress components to be introduced for a more accurate description of material hardening behavior, as expressed in Equation (4):

(4) $$\alpha =\sum_{k=1}^{N}\alpha_{k}$$

where *N* is the total number of superposed backstress components. To ensure computational efficiency while maintaining high fitting accuracy for the cyclic hysteresis curves, *N* = 3 was adopted in this study.

**Figure 9:**
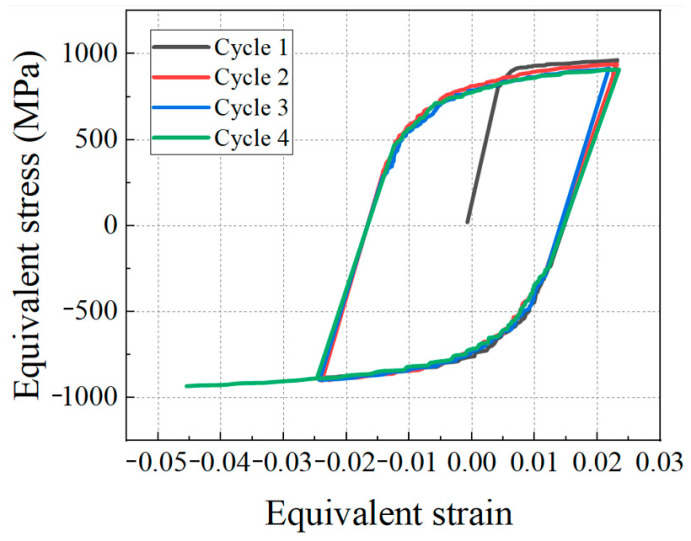
Experimental data from repeated cyclic shear tests.

Based on the above formulation, the parameters to be identified in the Chaboche mixed hardening model include *c*, *γ*, *Q* and *b*′. The cyclic loading data used for parameter identification were obtained from repeated cyclic shear tests, and the corresponding stress–strain curves are shown in Figure 9.

The Chaboche mixed hardening model describes hardening as a combination of kinematic and isotropic hardening. The first step is to determine the kinematic hardening parameters. In order to determine $C_{i}$($C_{i}=\gamma_{i}\cdot r_{i}$) and $\gamma_{i}$, the stress–inelastic strain curve can be obtained by removing the effect of isotropic hardening [32,33] from the monotonic stretching line of Q890 steel. Equations (5)–(7) are used.

(5) $$\gamma^{\left(i\right)}=\frac{1}{\varepsilon^{p\left(i\right)}}$$

(6) $$r^{\left(i\right)}=\left(\frac{\sigma^{\left(i\right)}-\sigma^{\left(i-1\right)}}{\varepsilon^{p\left(i\right)}-\varepsilon^{p\left(i-1\right)}}-\frac{\sigma^{\left(i+1\right)}-\sigma^{\left(i\right)}}{\varepsilon^{p\left(i+1\right)}-\varepsilon^{p\left(i\right)}}\right)\varepsilon^{p\left(i\right)}\left(i\neq 1\right)$$

(7) $$\sum_{i=1}^{3}r^{\left(i\right)}+\sigma_{0}=\sigma_{\max}$$

When determining the isotropic hardening parameters, the kinematic hardening variable must be decoupled to obtain the stress corresponding exclusively to isotropic hardening, denoted as σ*_i_*^0^, along with the corresponding accumulated plastic strain, denoted as *ε_i_^pl^*. Because the cyclic loading test consists of four cycles, four sets of (σ*_i_*^0,^ *ε_i_^pl^*) data points are obtained. The term *r*(*p*) in Equation (8) represents the difference between σ*_i_*^0^ and the initial yield stress, where *p* is equivalent to *ε_i_^pl^*, and the formulation of *r*(*p*) must incorporate the initial yield stress. Based on the experimental data, the parameter *Q* is calculated to be −190 MPa. The parameter *b*′ can then be determined through least-squares fitting. Therefore, the expression for the isotropic hardening parameters is given as follows:

(8) $$r\left(p\right)=-190\left(1-e^{-0.85p}\right)$$

The calibrated Chaboche mixed hardening parameters are listed in Table 3 and were used for the four-point bending finite element analysis and representative biaxial stress-state selection.

**Figure 10:**
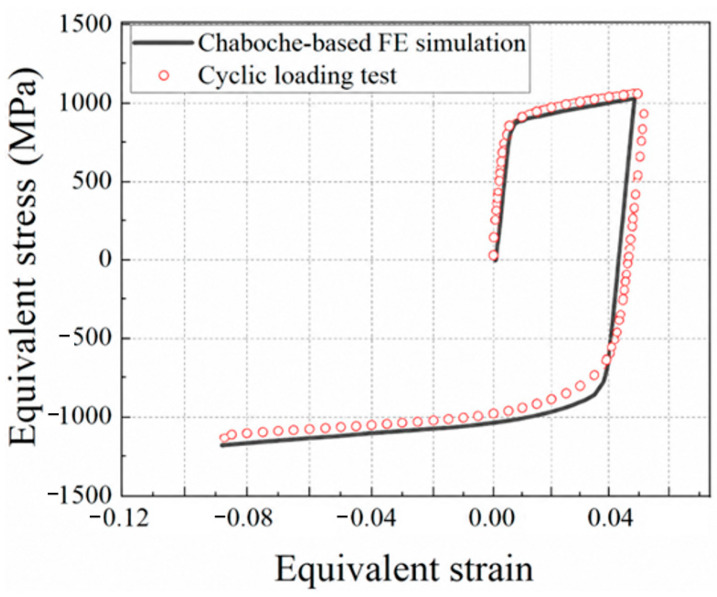
Comparison between cyclic shear test and Chaboche-based FE simulation.

To validate the accuracy of the calibrated Chaboche mixed hardening parameters, a cyclic loading model of a single element was established in ABAQUS/Standard version 2021. The simulation results obtained using the calibrated Chaboche parameters were compared with the experimental cyclic loading curves, as shown in Figure 10. As shown in Figure 10, the finite element curve obtained with the calibrated Chaboche parameters agrees well with the experimental cyclic loading curve in terms of the overall hysteresis shape, yield transition, and main stress response. The simulated initial yield strength matches the experimental value near 890 MPa (error ≈0.5%), and the maximum forward flow stress is well predicted at approximately 1030 MPa, with a minor relative error of 1.9% compared to the experimental 1050 MPa. However, some deviations remain during local reverse loading. The experimental data exhibits an early Bauschinger effect with a compression yield strength of approximately 730 MPa. In contrast, the Chaboche model slightly delays the reverse yielding, predicting a reverse yield strength of approximately 785 MPa (a relative deviation of ~7.5%). This indicates that the calibrated Chaboche mixed hardening model can reasonably characterize the elastoplastic response and Bauschinger effect of Q890 high-strength steel under cyclic loading. Some deviations remain during local reverse loading, suggesting that the model still simplifies the description of transient hardening and local softening. Nevertheless, its overall accuracy is sufficient for subsequent cyclic four-point bending simulations and representative biaxial stress-state construction.

#### 2.2.3. Four-Point Bending FE Modeling and Representative Condition Selection

**Figure 11:**
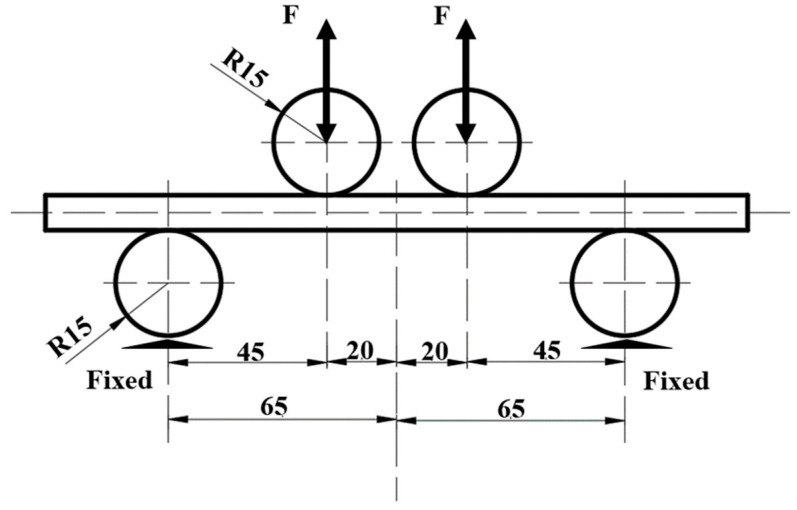
Schematic of the four-point bending fixture.

After obtaining the Chaboche mixed hardening parameters for Q890 high-strength steel, a four-point bending finite element (FE) model was established to construct and quantify representative in-plane biaxial stress states on the target surface. The model was used to analyze the effect of the specimen width-to-thickness ratio on the transverse stress $\sigma_{11}$, longitudinal stress $\sigma_{22}$, and biaxial stress ratio $\alpha_{t}$, and to determine the representative conditions for subsequent cyclic four-point bending tests. Based on the fixed specimen length and width defined in Section 2.1, different width-to-thickness ratios were obtained by varying the specimen thickness. The punch loading span and die support span of the four-point bending fixture were 40 mm and 130 mm, respectively, and the roller diameter was 30 mm. The fixture configuration and key geometric parameters are shown in Figure 11. This configuration provides a stable quasi-pure bending region between the two loading points, ensuring a consistent mechanical environment for constructing target-surface stress states in both simulations and experiments.

**Figure 12:**
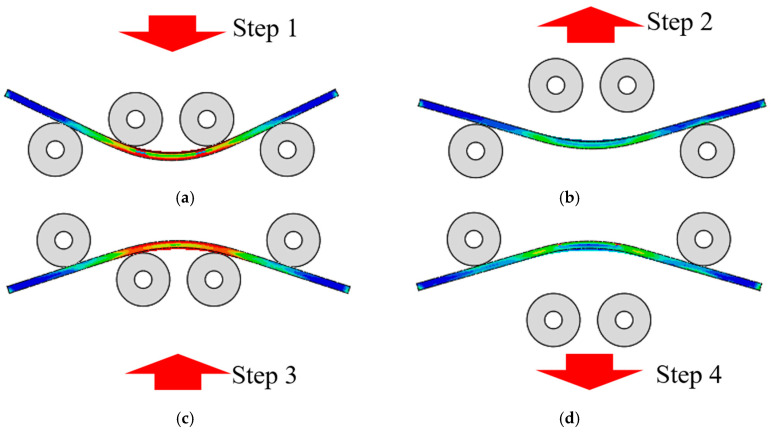
Schematic of the cyclic four-point bending procedure. (**a**) Forward bending. (**b**) Forward springback. (**c**) Reverse bending. (**d**) Reverse springback.

For the cyclic process involving forward/reverse bending and springback, an explicit dynamic finite element model was developed in ABAQUS/Explicit. The sheet was discretized using S4R elements with enhanced hourglass control, while the upper and lower rollers were modeled as rigid cylinders with the same dimensions as the actual tooling. Surface-to-surface contact was defined between the tooling and the sheet. The displacement-controlled numerical procedure strictly followed the sequence of forward loading, unloading springback, reverse loading, and reverse unloading springback, as shown in Figure 12. The tooling interfaces were modeled using surface-to-surface contact, with the friction coefficient set to 0.12. The boundary conditions were defined to match the cyclic physical process. In the forward loading pass, the lower forward-bending rollers were completely fixed, and a downward displacement in the Z direction was applied to the upper forward-bending rollers, while the reverse-bending rollers remained non-contacting. Conversely, in the reverse loading pass, the lower reverse-bending rollers were fixed, an upward displacement in the Z direction was applied to the upper reverse-bending rollers, and the forward-bending rollers were completely detached from contact.

To ensure numerical accuracy and computational efficiency, a mesh convergence analysis was conducted on the ABAQUS/Explicit four-point bending model. Comparing mesh sizes from 3 mm to 1 mm, a size of 1.2 × 1.2 × 1.2 mm was ultimately adopted. This resolution successfully captured the through-thickness bending stress gradient and stabilized the maximum equivalent stress (variation <0.6% compared to the 1 mm mesh) without incurring excessive computational costs. To ensure the reliability of the quasi-static explicit analysis, a conservative mass scaling approach was utilized. Energy balance checks confirmed that the kinetic energy remained negligible during plastic deformation, and the artificial hourglass energy was strictly controlled below the 5% acceptable threshold.

**Figure 13:**
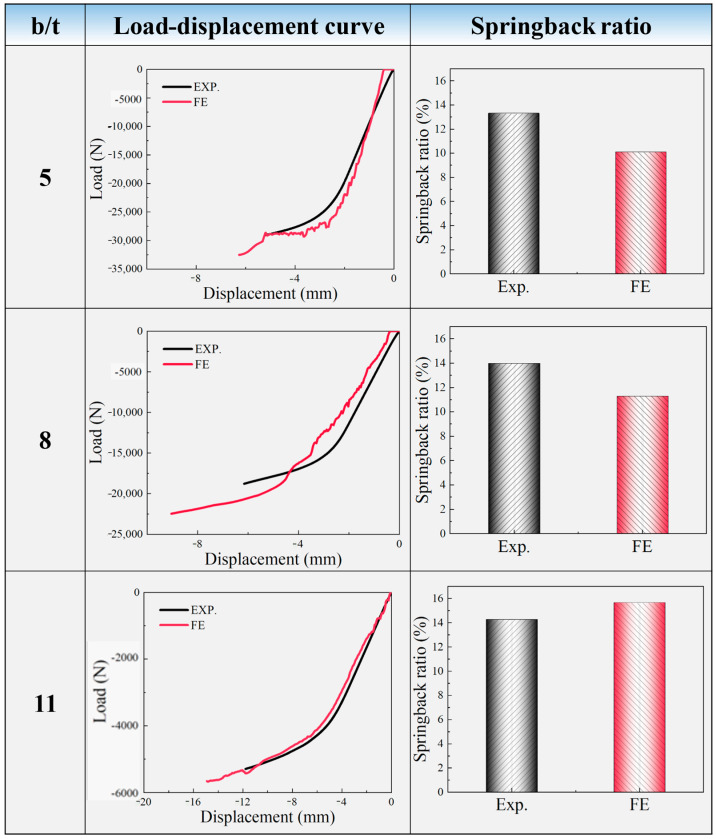
Comparison of experimental force–displacement curves and springback angles under different width-to-thickness ratios between experimental and simulation tests.

To verify the reliability of the established finite element model, its numerical predictions were compared with the physical experimental data (the detailed experimental procedures and setups are presented in Section 3). Figure 13 presents the comparison between the experimental and simulated force–displacement curves and springback angles under different width-to-thickness ratios (b/t = 5, 8, and 11). The results indicate that the force–displacement curves simulated by the finite element model exhibit good consistency with the experimental data. Despite the slight numerical oscillations inherent to the explicit dynamic solving scheme at larger deformations, the numerical model successfully captured the key nonlinear deformation characteristics, accurately reproducing the macroscopic yield point and the subsequent hardening trajectory during the bending process. To quantitatively evaluate the accuracy of the springback prediction, the specific deviations were calculated. Although the finite element model slightly underestimated the springback ratio for the relatively thicker specimens, the absolute deviations were approximately 3.3% for b/t = 5 (Exp: ~13.4%, FE: ~10.1%) and 2.7% for b/t = 8 (Exp: ~14.0%, FE: ~11.3%). Conversely, for the thinner specimen (b/t = 11), the model exhibited a slight overestimation, with an absolute deviation of only 1.4% (Exp: ~14.3%, FE: ~15.7%). The overall deviation was strictly maintained within acceptable engineering tolerances. This confirms that the numerical model can reliably simulate the macroscopic physical deformation and the path-dependent Bauschinger effect of the Q890 high-strength steel heavy plates, laying a valid foundation for the subsequent stress-state extraction.

**Figure 14:**
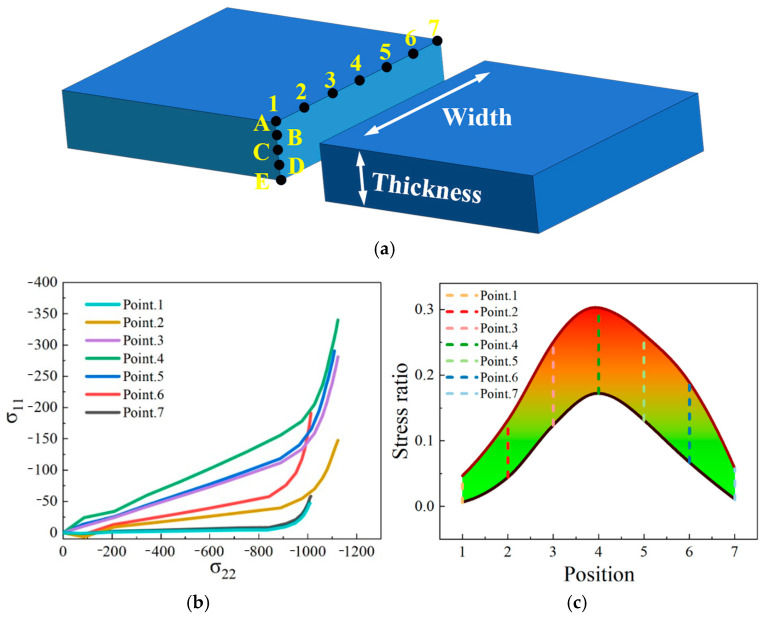
With a width-to-thickness ratio of 17, stress changes during positive bending at different width positions on the upper surface: (**a**) Specific locations along the width and thickness directions; (**b**) σ11-σ22 curves at different width positions; (**c**) Stress ratio ranges at different width positions.

Based on the above model, four-point bending FE analyses were further conducted under different width-to-thickness ratios. Due to edge effects, the stress ratio decreases near the boundaries of the blank but maintains a stable biaxial stress state in the central region, as illustrated in Figure 14. Therefore, the stress components are extracted from the center of the specimen’s inner surface to authentically represent the actual forming conditions. The “inner surface” is defined as the specific surface that directly contacts the upper die during the initial forward bending phase. By extracting the stress tensor components $\sigma_{11}$ and $\sigma_{22}$ from the inner-surface elements along the centerline of the plate deformation zone, the biaxial stress ratio was defined as given in Equation (9) and used as the key indicator for quantifying the in-plane stress state:

(9) $$\alpha_{t}=\frac{\sigma_{11}}{\sigma_{22}}$$

**Figure 15:**
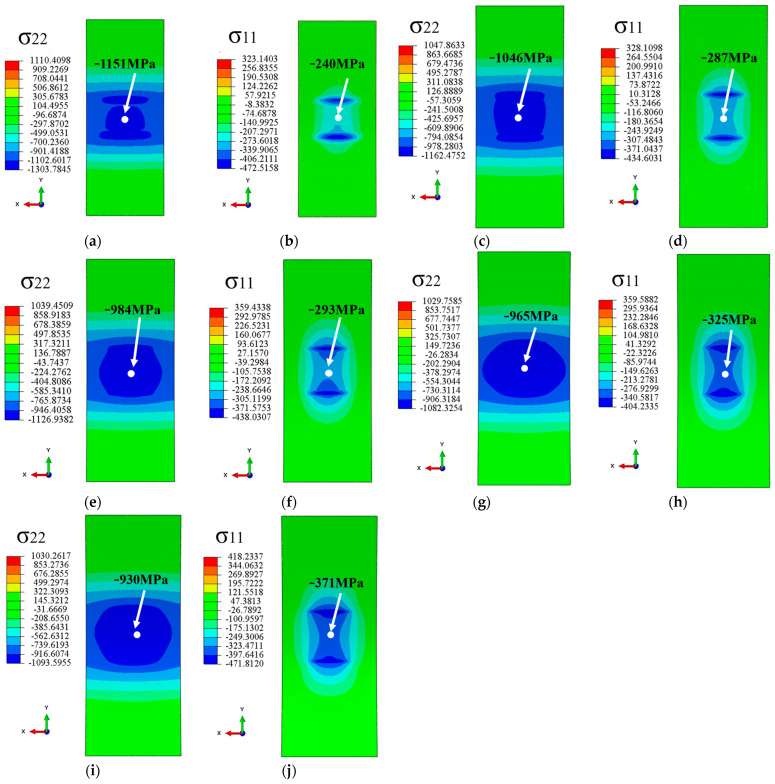
Simulated stress contour plots of the target surface under different width-to-thickness ratios. (**a**,**b**) b/t = 5, t = 16 mm. (**c**,**d**) b/t = 8, t = 10 mm. (**e**,**f**) b/t = 11, t = 7.3 mm. (**g**,**h**) b/t = 15, t = 5.4 mm. (**i**,**j**) b/t = 17, t = 4.7 mm.

The simulated stress contour plots under different width-to-thickness ratios are shown in Figure 15. The results show that the target surface is not subjected to a uniaxial stress state. Instead, a transverse stress component $\sigma_{11}$ is generated together with the longitudinal stress $\sigma_{22}$, forming an in-plane biaxial stress state on the target surface. To quantify this biaxial stress-state difference, $\sigma_{11}$ and $\sigma_{22}$ were extracted from the inner-surface elements along the centerline of the quasi-pure bending region, and the biaxial stress ratio was calculated as $\alpha_{t}=\sigma_{11}/\sigma_{22}$. 

The specimens with thicknesses of 16, 10, 7.3, 5.4, and 4.7 mm can, to a certain extent, modulate the dynamic evolution intervals of the multiaxial stress ratio on the target surface by altering the degree of transverse constraint. Therefore, these specimens with different width-to-thickness ratios are utilized as experimental variables to approximately cover various gradients of in-plane biaxial stress states in the subsequent cyclic four-point bending tests. These results indicate that, although the multiaxial stress evolution in actual heavy-plate forming is synergistically governed by multiple complex mechanical factors, within the current testing framework, the width-to-thickness ratio can serve as an effective macroscopic geometric tuning parameter to approximately construct the dynamic multiaxial stress conditions experienced during the actual pressing process.

### 2.3. Cyclic Four-Point Bending Test and Stress Correction

**Figure 16:**
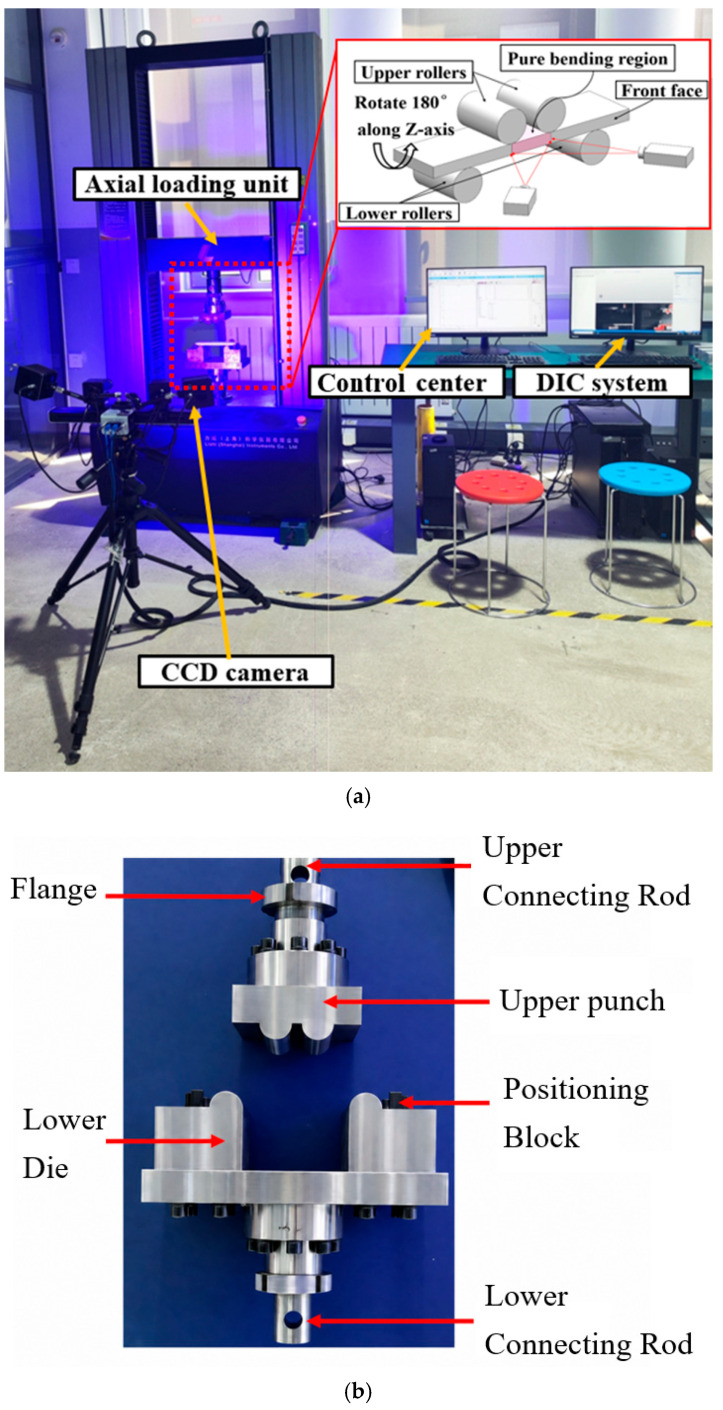
Physical setup of the four-point bending test system and fixture. (**a**) Four-point bending test system. (**b**) Four-point bending fixture.

To characterize the Bauschinger effect of Q890 high-strength steel under different in-plane stress states, cyclic four-point bending was employed as a testing method to construct stress paths on the target surface. The cyclic four-point bending test system shown in Figure 16 consists of a bending fixture, a universal testing machine (LD26.105, LI-SHI (Shanghai) Scientific Instruments Co., Ltd., Shanghai, China), and a digital image correlation (DIC) system (XTDIC-CONST-HR, XTOP 3D Technology (Shenzhen) Co., Ltd., Shenzhen, China) for real-time full-field strain measurement on the target surface. To ensure measurement fidelity, the DIC equipment was rigorously calibrated by a professional certification agency prior to the experiments. The specimen dimensions and testing schemes are listed in Table 4.

In this study, one loading pass refers to one forward or reverse bending step followed by unloading and springback, while one complete loading cycle consists of one forward-bending pass and the subsequent reverse-bending pass. During each loading pass, the target-surface strain was controlled by the displacement of the upper loading rollers. The target-surface pre-strains were set to 0.02, 0.03, 0.04, and 0.05 (see Table 4), covering the typical strain range encountered in forming large-scale double-curvature thick plates. After the forward-bending pass, the specimen was flipped by 180° to perform reverse bending on the same target surface. The roller–sheet contact regions were coated with white petrolatum to reduce friction. All tests were performed under displacement control mode at a constant loading rate of 2 mm/min. Each experimental condition was independently repeated three times. After confirming the fundamental consistency of the test results, the average values were adopted for subsequent analysis.

The total load P applied by the upper loading rollers was synchronously recorded by the load sensor of the universal testing machine and was converted into the external bending moment M in the quasi-pure bending region according to the four-point bending force relationship. Assuming that the two upper loading forces were approximately equal, the external bending moment in the quasi-pure bending region was calculated as M=Pa/2, where a is the horizontal distance between the lower support roller and the adjacent upper loading roller. The stress response of the target surface was then inversely determined by combining the target surface strain measured by DIC with the neutral-layer correction method described below.

**Figure 17:**
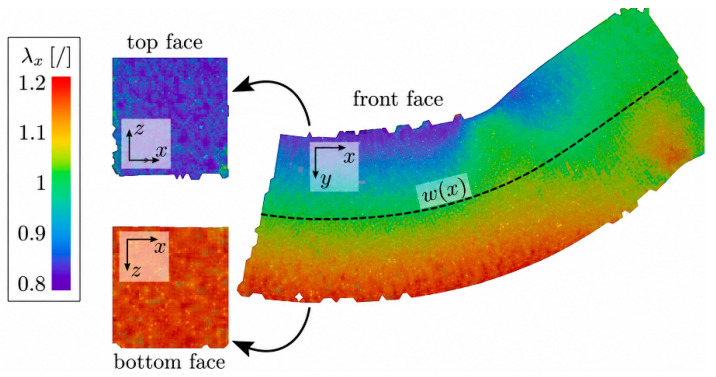
Strain distribution and measurement region of the bending specimen, adapted from [34].

The DIC system was used to capture the full-field displacement and strain evolution of the target surface in the quasi-pure bending region, with particular emphasis on the surface strain distribution along the longitudinal direction, as shown in Figure 17. The stress correction was then applied to convert the surface strains and bending moments into the equivalent stress–strain responses of the target surface. This combined DIC-plus-correction approach enabled systematic acquisition of equivalent stress–strain curves under various biaxial stress ratios over multiple cycles. The related derivation is given as follows.

Under pure bending, the stress through the specimen thickness gradually changes from tensile to compressive, and the position where the stress becomes zero is defined as the neutral layer. In ideal elastic bending, the neutral layer coincides with the section centroid. However, once bending enters the plastic stage, the stress distributions on the tensile and compressive sides become asymmetric, causing the neutral layer to deviate from the centroid and shift toward the compressive side. If the surface stress is still back-calculated using the elastic bending formula under the assumption that the neutral layer remains at the centroid, the actual bending stress after plastic deformation will be overestimated. Therefore, it is necessary to correct the neutral-layer position based on the measured surface strain. The related derivation is given as follows [34,35]:

The corresponding strains and stresses on the top and bottom surfaces of the specimen are denoted by $\varepsilon_{t}$, $\varepsilon_{c}$, $\sigma_{t}$ and $\sigma_{c}$, respectively. The instantaneous position of the neutral axis is located at distances $h_{t}$ and $h_{c}$ from the tensile and compressive surfaces, respectively, and the total thickness h is given by the sum of $h_{t}$ and $h_{c}$. According to Timoshenko’s assumption, a linear strain distribution through the specimen thickness is considered. Under pure bending, the neutral axis is determined by imposing the equilibrium condition that the resultant axial force vanishes. The second condition required for the solution is the equilibrium of the internal bending moment.

According to axial force equilibrium, the areas under the stress–strain curves on the tensile and compressive sides are equal, yielding:

(10) $$\sum_{j=1}^{m}\sigma_{tj}\Delta \varepsilon_{tj}=\sum_{j=1}^{m}\sigma_{cj}\Delta \varepsilon_{cj}$$

where $\sigma_{\mathrm{tj}}$ and $\sigma_{\mathrm{cj}}$ are the stresses on the tensile and compressive surfaces at the j-th strain increment, respectively; $\Delta \varepsilon_{\mathrm{tj}}$ and $\Delta \varepsilon_{\mathrm{cj}}$ are the corresponding strain increments on the tensile and compressive sides, respectively; m is the total number of increments up to the current loading state; and j denotes the increment index.

The equilibrium equation between the internal and external bending moments can be written as:

(11) $$M_{m}=\frac{bh_{tm}^{2}}{\varepsilon_{tm}^{2}}\sum_{j=1}^{m}\sigma_{tj}\varepsilon_{tj}\Delta \varepsilon_{tj}+\frac{bh_{cm}^{2}}{\varepsilon_{cm}^{2}}\sum_{j=1}^{m}\sigma_{cj}\varepsilon_{cj}\Delta \varepsilon_{cj}$$

where M_m_ is the applied bending moment at the m-th increment; b is the specimen width; $h_{\mathrm{tm}}$ and $h_{\mathrm{cm}}$ are the distances from the neutral layer to the tensile and compressive surfaces at the m-th increment, respectively; $\varepsilon_{\mathrm{tm}}$ and $\varepsilon_{\mathrm{cm}}$ are the strains on the tensile and compressive surfaces at the m-th increment, respectively; and $\varepsilon_{\mathrm{tj}}$ and $\varepsilon_{\mathrm{cj}}$ are the strains on the tensile and compressive sides at the j-th increment, respectively.

Based on the linear strain distribution through the thickness, the following relation can be obtained:

(12) $$\frac{bh_{t}^{2}}{\varepsilon_{t}^{2}}=\frac{bh_{c}^{2}}{\varepsilon_{c}^{2}}=\frac{bh^{2}}{{\left(\varepsilon_{t}+\varepsilon_{c}\right)}^{2}}$$

where $h_{t}$ and $h_{c}$ are the distances from the neutral layer to the tensile and compressive surfaces, respectively; h is the total specimen thickness; and $\varepsilon_{t}$ and $\varepsilon_{c}$ are the strains on the tensile and compressive surfaces, respectively.

Thus, for each increment j, the geometric relationship between the strain increments on the tensile and compressive sides can be derived as follows:

(13) $$\sigma_{tj}\Delta \varepsilon_{tj}=\sigma_{cj}\Delta \varepsilon_{cj}$$

This indicates that the total strain energy stored or dissipated on the tensile and compressive sides is equal. Therefore, Equation (11) can be rewritten as:

(14) $$M_{m}=\frac{bh^{2}}{{\left(\varepsilon_{tm}+\varepsilon_{cm}\right)}^{2}}\sum_{j=1}^{m}\left[\sigma_{tj}\varepsilon_{tj}(\varepsilon_{cj}+\varepsilon_{tj})\right]$$

Once the stresses and strains from increment 1 to *k* − 1 are determined from DIC measurements, the equivalent stress at any increment *k* can be obtained from Equation (15):

(15) $$\sigma_{kt}=({\bar{M}}_{K}-\sum_{j=1}^{m}\sigma_{tj}\varepsilon_{tj}\Delta \varepsilon_{tj})/(\varepsilon_{k}\Delta \varepsilon_{kt})$$

where ${\bar{M}}_{K}=M_{k}/[\frac{bh^{2}}{{\left(\varepsilon_{tm}+\varepsilon_{cm}\right)}^{2}}]$, ${\bar{\varepsilon }}_{k}=(\varepsilon_{ck}+\varepsilon_{tk})$.

The tensile and compressive stresses can then be calculated using any two consecutive increments, k − 1 and k, as follows:

(16) $$\sigma_{sk}=\frac{{\bar{M}}_{K}-{\bar{M}}_{K-1}}{\bar{\varepsilon }\Delta \varepsilon_{sk}}$$

where s = t denotes the tensile stress and s = c denotes the compressive stress. The above equations give the stress at any increment k = 1, …, m.

**Figure 18:**
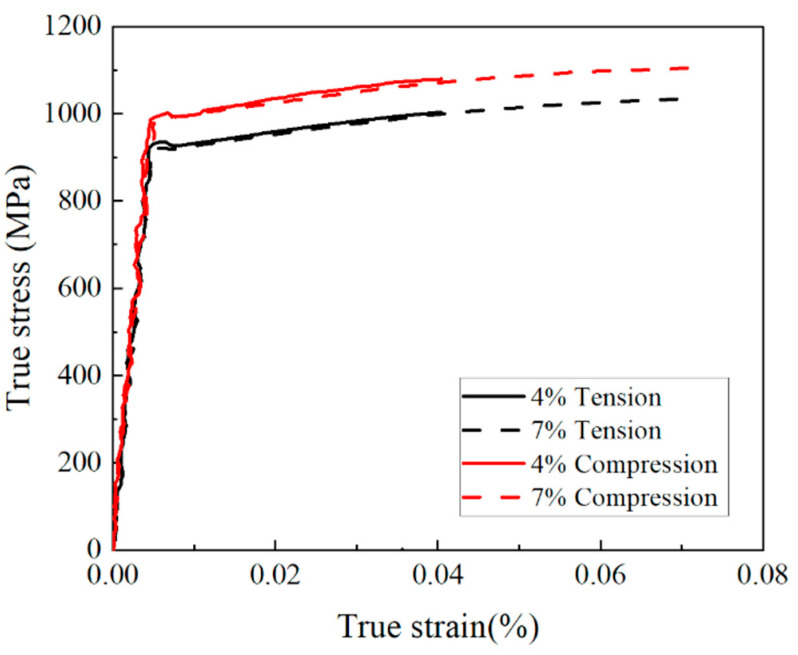
Flow stress–strain curves of tension and compression tests.

**Figure 19:**
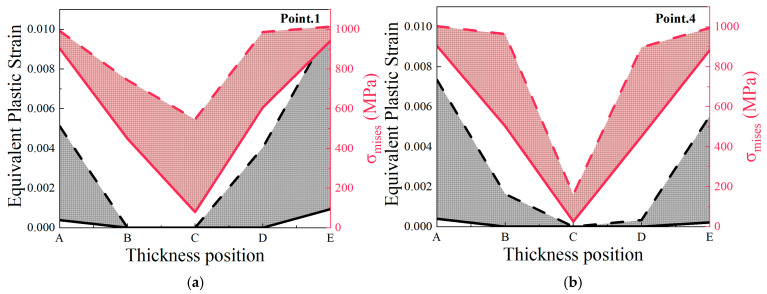
Equivalent plastic strain and Mises stress changes during positive bending plastic deformation at different width positions on the side surface (point 1) and the middle position (point 4) with a width-to-thickness ratio of 17. The solid line represents the beginning of plastic deformation, and the dashed line represents the end of bending. The red color represents the Mises stress, and the gray color represents the equivalent plastic strain. (**a**) Point 1. (**b**) Point 4.

The above equations provide a rigorous discrete incremental framework that accounts for the potentially asymmetric tension–compression behavior. However, Figure 18 presents the flow stress–strain curves obtained from the uniaxial tension and compression tests. As analyzed from the figure, although the investigated Q890 steel exhibits a slight deviation in the initial yield strength between tension and compression, its subsequent plastic work-hardening rates demonstrate a high degree of consistency and parallelism. Furthermore, Figure 19 presents the numerical distributions of the equivalent plastic strain and von Mises stress along the thickness direction at different width positions during the bending deformation. The numerical analysis further confirms that the plastic neutral layer—where the equivalent plastic strain and stress reach their minimum thresholds (near position C)—maintains a stable geometric depth across the entire width of the specimen, with no significant spatial migration occurring during the deformation process. Under such conditions of macroscopic material symmetry and negligible neutral layer offset, the classical Nadai correction method offers a highly efficient analytical equivalent to the discrete framework. Consequently, the corrected surface longitudinal normal stress can be directly derived by introducing the instantaneous slope of the bending moment-strain curve:

(17) $$\sigma_{s}^{N}\;=\frac{2}{bt^{2}}\;(2M+\frac{dM}{d\varepsilon_{s}})$$

where *b* is the specimen width, *t* is the thickness, *M* is the bending moment in the pure bending region, and *dM*/$d\varepsilon_{s}$ denotes the instantaneous rate of change in the moment-strain curve.

To compare the bending test results with the shear test results on a unified basis, the target-surface stress–strain data corrected by the neutral-layer method were converted into equivalent values according to the von Mises criterion. Considering that the surface layer in the quasi-pure bending region approximately satisfies the plane-stress condition and that the in-plane shear stress can be neglected, the equivalent stress of the target surface can be expressed as:

(18) $$\bar{\sigma }=\sqrt{\sigma_{11}^{2}-\sigma_{11}\sigma_{22}+\sigma_{22}^{2}}$$

where $\sigma_{11}$ and $\sigma_{22}$ are the surface normal stresses in the width and length directions of the specimen, respectively.

Similarly, under the plane-stress condition and assuming plastic incompressibility, the equivalent strain of the target surface can be expressed as:

(19) $$\bar{\varepsilon }=\frac{2}{\sqrt{3}}\sqrt{\varepsilon_{11}^{2}+\varepsilon_{11}\varepsilon_{22}+\varepsilon_{22}^{2}}$$

**Figure 20:**
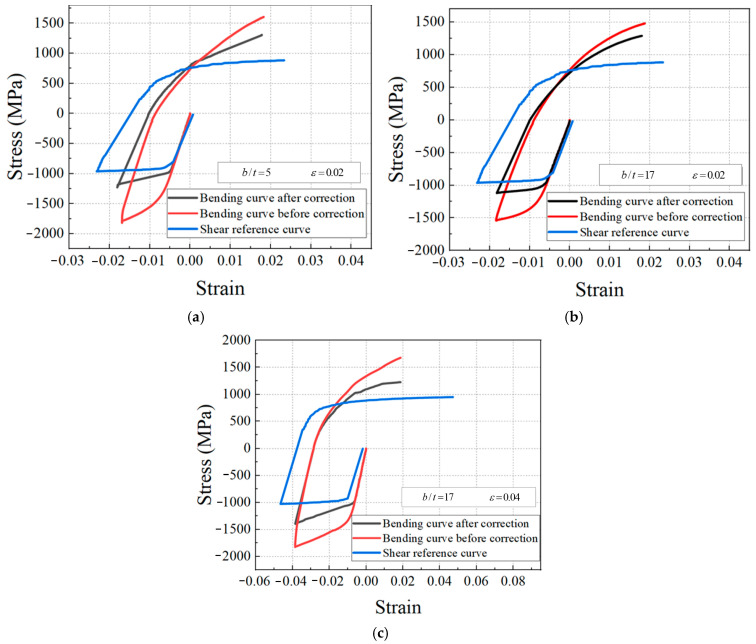
Comparison of equivalent stress–strain curves obtained from bending and shear tests before and after neutral-layer correction. (**a**) b/t = 5, ε = 0.02. (**b**) b/t = 17, ε = 0.02. (**c**) b/t = 17, ε = 0.05.

Figure 20 compares the equivalent stress–strain curves before and after neutral-layer correction with the shear reference curve under three representative conditions. Before correction, the bending stress–strain curves were significantly higher than the shear curve in the plastic stage, indicating that direct back-calculation under the assumption that the neutral layer coincides with the section centroid overestimates the target-surface stress. After correction, the bending curves converged toward the shear curve and showed good agreement in the small-strain stage. At larger strains, the corrected bending curves remained slightly higher than the shear curve, suggesting that the target surface was not subjected to a pure shear state but was jointly affected by longitudinal bending deformation, width-direction constraint, and through-thickness stress gradients. Overall, the neutral-layer correction reduced the influence of bending geometry and surface-position effects on stress characterization, making the obtained equivalent stress–strain curves more suitable for subsequent extraction of Bauschinger-effect parameters.

**Figure 21:**
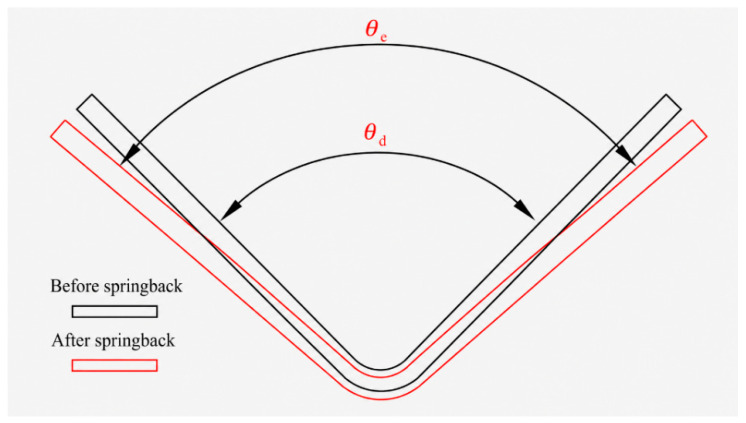
Schematic of springback.

Since the pre-strain during cyclic bending affects the subsequent reverse-yielding response, flow stress, and unloading behavior, three types of variables were systematically studied: (i) biaxial stress ratio $\alpha_{t}$, (ii) loading cycle number (1st to 4th cycle), and (iii) target-surface pre-strain. During forward and reverse bending, the same physical surface was consistently used for analysis. This surface served as the inner surface during forward bending and became the outer surface after the specimen was flipped by 180° for reverse bending; it is defined here as the “target surface.” To quantitatively characterize the springback behavior and the corresponding target-surface response under different conditions, the springback ratio was introduced as a macroscopic evaluation index. A schematic of springback is shown in Figure 21. Considering the operational inconvenience of frequently using a 3D scanner during continuous experiments, this study employs the DIC image method to measure the springback angle. Specifically, high-resolution DIC images captured after unloading are imported into AutoCAD 2021 software, and the same image is measured three times to obtain an average value. This method effectively reduces random errors caused by manual operations, ensuring the reliability of the experimental data.

The springback ratio K was used to characterize the degree of springback:

(20) $$K=\frac{\theta_{e}-\theta_{d}}{\theta_{d}}\times 100\%$$

where $\theta_{e}$ is the angle after springback, $\theta_{d}$ is the angle before springback.

To ensure the quantitative reliability of the experimental results, the measurement uncertainties were systematically evaluated and are summarized in Table 5. According to the metrological verification, the load measurement error of the testing machine is within ±0.5%, and the relative expanded uncertainty of the DIC system for displacement measurement is $U_{rel}=0.3\%$ (k=2). The uncertainty of the springback angle, evaluated via repeated image-based extractions, is approximately ±0.2°. Consequently, the uncertainty of the extracted stress—including the initial yield stress and compression yield stress—is estimated to be within ±2.0%. The uncertainties of the Bauschinger-related parameter (such as BSP) were determined through error propagation, showing that the measurement scatter is on the order of $10^{-2}$. These quantified uncertainties indicate that while experimental scatter may slightly influence the absolute numerical values, the overall comparative trends discussed in this study remain robust.

## 3. In-Plane Biaxial Stress-State-Dependent Cyclic Plasticity and Springback Evolution

### 3.1. Effect of Biaxial Stress Ratio on Bauschinger Effect

**Figure 22:**
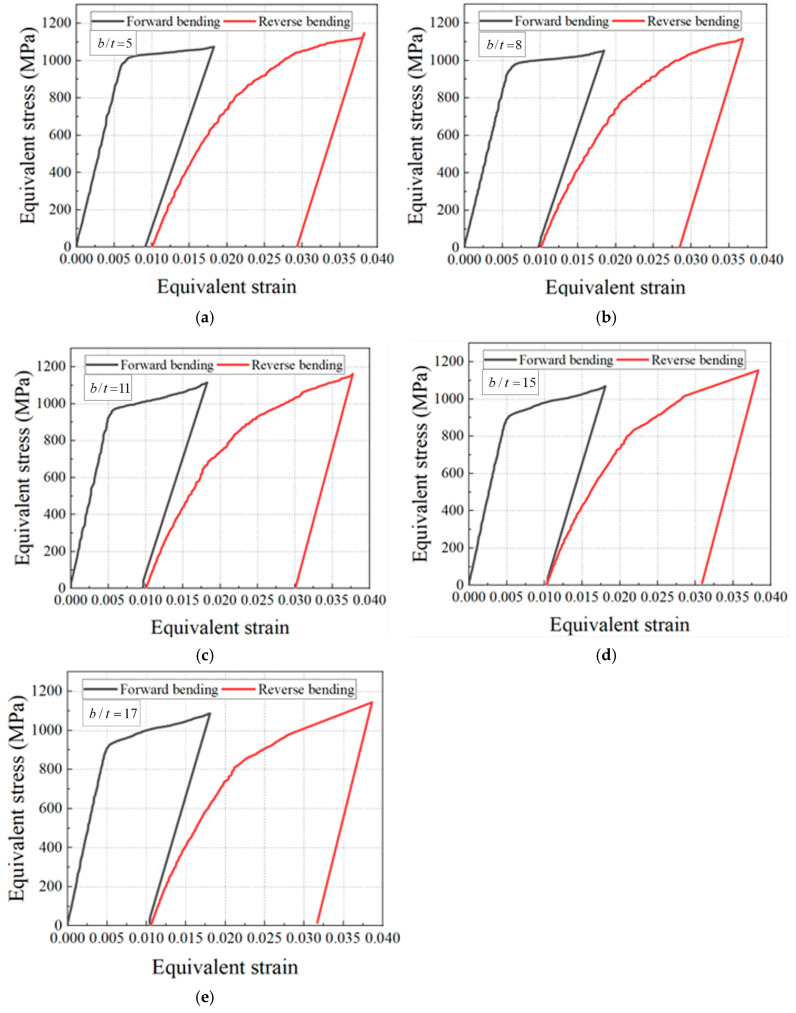
Target-surface equivalent stress–strain curves under different stress states during forward and reverse bending. (**a**) b/t = 5. (**b**) b/t = 8. (**c**) b/t = 11. (**d**) b/t = 15. (**e**) b/t = 17.

**Figure 23:**
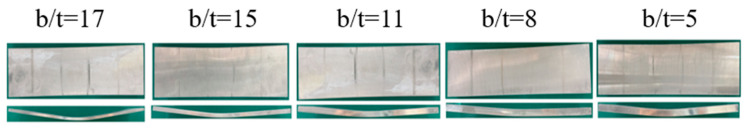
Specimen images of specimens with different width-to-thickness ratios under a strain of 0.02.

Figure 22 shows the equivalent stress–strain curves of the target surface under different in-plane stress states characterized by the thickness-to-width ratio. All conditions exhibit clear elastoplastic loading, unloading, and reverse-loading responses, with premature yielding during reverse loading, indicating a pronounced Bauschinger effect in Q890 high-strength steel during cyclic four-point bending. Figure 23 shows the experimental results for specimens with different width-to-thickness ratios under a target-surface strain of 0.02. As b/t increases from 5 to 17, the forward loading stress level decreases overall, while the reverse yield position and reverse flow stress also change. This indicates that the Bauschinger effect is not only governed by loading-path reversal, but also depends on the biaxial stress state characterized by $\alpha_{t}$. Since the curves are relatively close to each other, the differences among different stress states cannot be fully distinguished from the stress–strain curves alone.

To quantitatively characterize the effect of the stress state on cyclic stress asymmetry, the initial yield stress $\sigma_{Y}$, forward maximum flow stress $\sigma_{f}$, and reverse yield stress $\sigma_{r}$, were extracted, as listed in Table 6. The 0.2% plastic strain offset criterion was strictly employed to identify the initial yield stress and the reverse yield stress. The Bauschinger ratio B, Bauschinger hardening parameter BHP, Bauschinger stress parameter BSP, and Bauschinger energy parameter BEP were then calculated using Equations (21)–(24) [36,37,38,39].

B indicates the magnitude of the Bauschinger effect, but fails to account for the flow stress evolution.

(21) $$B=\frac{\sigma_{y}-\sigma_{r}}{\sigma_{r}}$$

BSP reflects the relative reduction in reverse yield stress, with a higher value indicating a more pronounced Bauschinger effect.

(22) $$BSP=\frac{\sigma_{f}-\sigma_{r}}{\sigma_{f}}$$

BHP characterizes the ratio of reverse yield stress reduction to forward hardening extent, reflecting the change in the material’s hardening capacity during reverse loading.

(23) $$BHP=\frac{\sigma_{y}-\sigma_{r}}{\sigma_{f}-\sigma_{r}}$$

*BEP* quantifies the energy dissipated from the reverse yield strength reduction, with BEP > 1 confirming the effect’s presence.

(24) $$BEP=\frac{\sigma_{f}-\sigma_{r}}{\sigma_{f}-\sigma_{y}}$$

**Figure 24:**
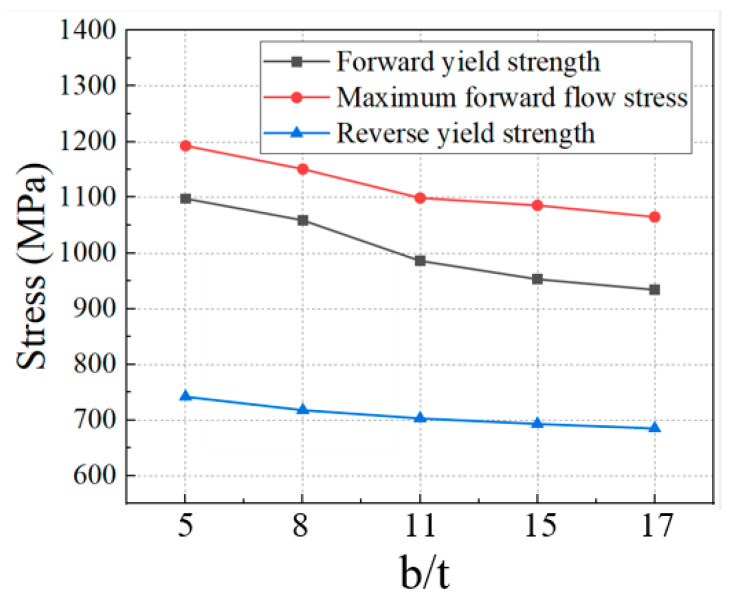
Comparison of characteristic stress parameters of the target surface under different stress states.

Figure 24 shows the characteristic stress parameters of the target surface under different stress states. As the corresponding b/t increased from 5 to 17, the initial yield strength decreased from 1098 MPa to 934 MPa, the maximum flow stress during forward loading decreased from 1193 MPa to 1065 MPa, and the reverse yield strength decreased from 742 MPa to 685 MPa. For all conditions, $\sigma_{f}$ > $\sigma_{Y}$ > $\sigma_{r}$, indicating that yielding occurred significantly earlier during reverse loading after forward bending, and that the material exhibited a pronounced Bauschinger effect. These results suggest that variations in the stress state affect the stress of the target surface and the reverse-loading response.

**Figure 25:**
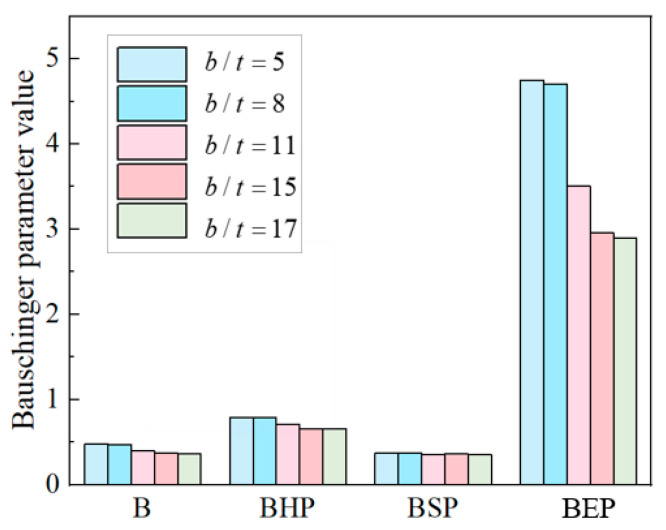
Bauschinger-related parameters under different in-plane stress states.

Figure 25 and Table 7 show the variations in Bauschinger-related parameters of the target surface under different stress ratios. As the b/t increased from 5 to 17, B, BHP, and BEP generally decreased. Among them, BEP decreased from 4.747 to 2.900, indicating its high sensitivity to stress-state variations. B and BHP decreased from 0.47978 and 0.789 to 0.363 and 0.655, respectively, suggesting that the asymmetry between forward and reverse loading responses weakened with increasing stress ratio. In contrast, BSP showed only a slight change, decreasing from 0.378 to 0.356 with minor fluctuations among different stress ratios, indicating its relatively low sensitivity to stress-ratio variation. Overall, the in-plane stress state affects the reverse-loading response of Q890 high-strength steel during cyclic bending.

**Figure 26:**
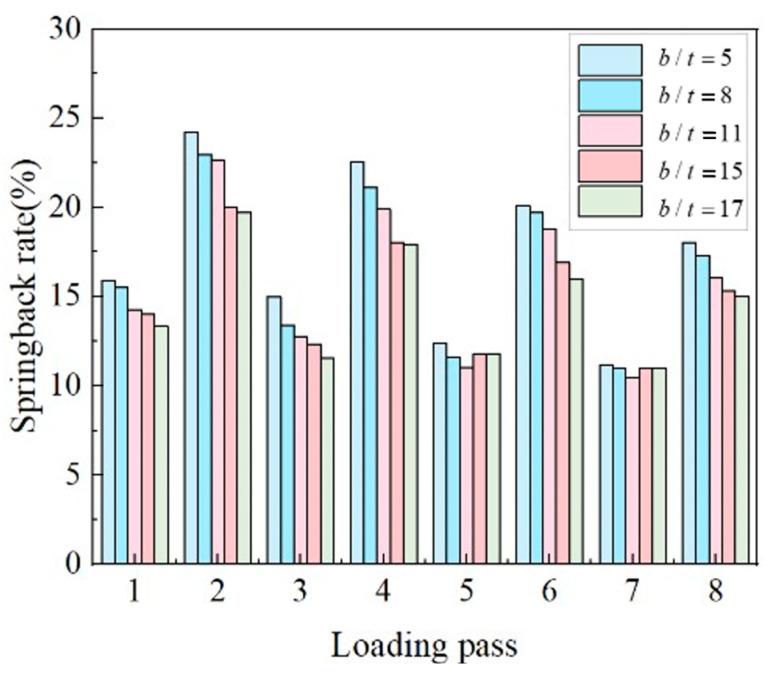
Variation in springback ratio with loading pass under different in-plane stress states.

Based on the above analysis, the variation in springback ratio with loading pass was further examined for specimens with different width-to-thickness ratios, as shown in Figure 26. Under different stress states, the springback ratio exhibited both a clear odd–even alternation (higher in even-numbered reverse-bending passes) and a gradual attenuation with increasing loading pass. The springback ratio in even-numbered passes, corresponding to the reverse path, was consistently higher than that in the adjacent odd-numbered passes, corresponding to the forward path, indicating a pronounced path-reversal effect during cyclic bending. As a result, the target surface exhibits a different through-section stress distribution before unloading, leading to a stronger tendency for elastic recovery. With increasing loading passes, the springback ratio under all stress states decreased overall and gradually stabilized. This is likely because plastic deformation and residual stress were continuously redistributed during cyclic loading, reducing the proportion of recoverable elastic deformation during unloading [40,41]. Further comparison among different stress ratios shows that the in-plane stress state has a more pronounced effect on reverse-bending springback. Reverse-bending springback generally decreased with increasing $\alpha_{t}$, whereas the forward-bending springback did not show a strictly monotonic dependence on $\alpha_{t}$, suggesting that variations in the in-plane biaxial stress state affect stress inheritance and springback response during cyclic bending.

### 3.2. Effect of Cyclic Loading History on Bauschinger Effect and Springback

**Figure 27:**
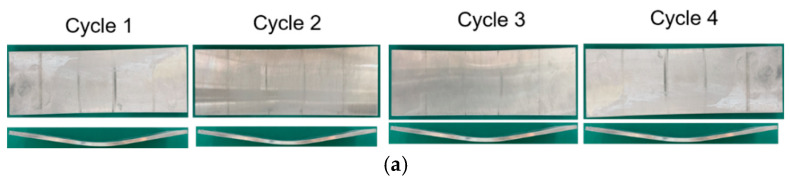

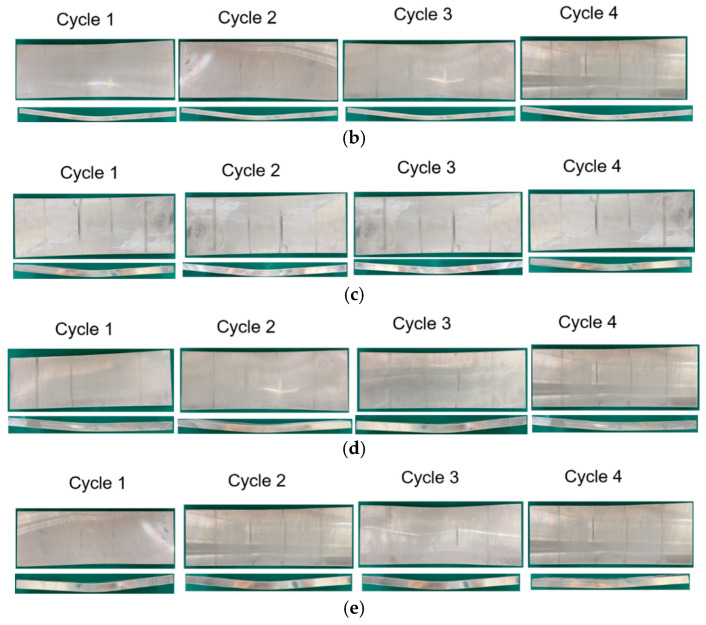
Experimental results of specimens under different loading cycles. (**a**) b/t = 17. (**b**) b/t = 15. (**c**) b/t = 11. (**d**) b/t = 8. (**e**) b/t = 5.

During cyclic bending, the target surface undergoes repeated forward and reverse loading as the number of loading cycles increases, and its response continuously evolves with the loading history. The experimental results for different loading cycles are shown in Figure 27.

**Figure 28:**
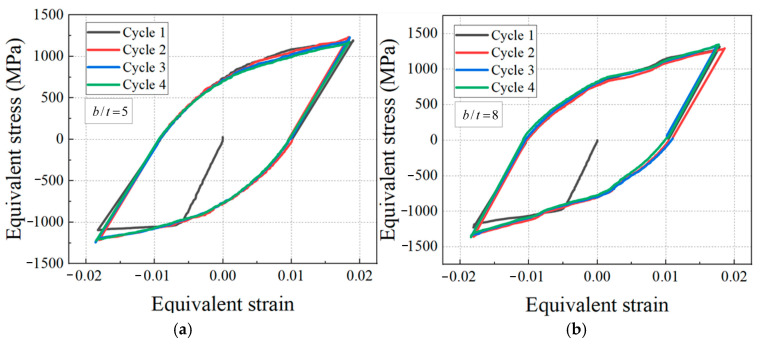

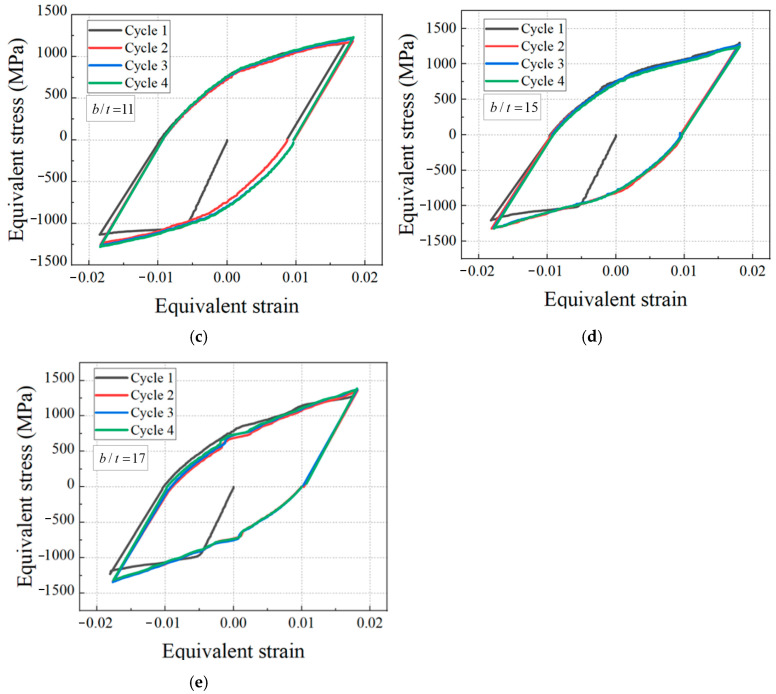
Equivalent stress–strain curves of the target surface under different loading cycles. (**a**) b/t = 5. (**b**) b/t = 8. (**c**) b/t = 11. (**d**) b/t = 15. (**e**) b/t = 17.

As shown in Figure 28, the equivalent stress–strain curves of the target surface evolve with increasing loading cycles under all biaxial stress ratios, indicating that the cyclic response is affected by loading history during repeated forward and reverse bending.

**Figure 29:**
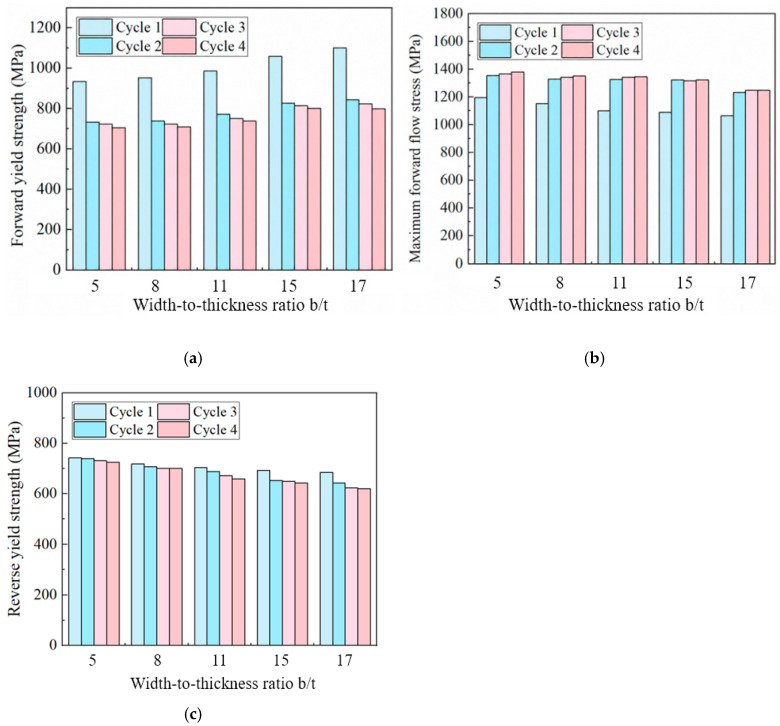
Evolution of target-surface characteristic stress parameters with loading cycles under different in-plane stress states. (**a**) The forward loading yield stress. (**b**) The reverse yield stress. (**c**) The maximum forward loading flow stress.

To further quantify the effect of cyclic history on the key response parameters of the target surface, characteristic stress parameters were extracted at different loading cycles, as listed in Table 8 and plotted in Figure 29. $\sigma_{Y}^{i}$ is the forward loading yield stress of the *i*-th cycle, $\sigma_{r}^{i}$ is the reverse yield stress of the *i*-th cycle, and $\sigma_{f}^{i}$ is the maximum forward loading flow stress of the *i*-th cycle. Under different in-plane stress states, these parameters show similar evolution trends with increasing loading cycles: the initial yield strength decreases markedly after the first cycle and then tends to stabilize; the maximum forward flow stress increases significantly in the second cycle; and the reverse yield strength continuously decreases with increasing cycles. This may be attributed to the repeated tension–compression reversal experienced by the target surface during cyclic bending, which promotes the accumulation of plastic deformation and modifies the stress response [42]. After the first cycle, the material remains in a rapid evolution stage following initial deformation; therefore, the second cycle exhibits a more pronounced hardening response than the first cycle. The relative stabilization observed as cyclic loading proceeds may be attributed to the gradual evolution of the dislocation structure and internal stress state from rapid adjustment to a more stable configuration [43,44,45].

Moreover, repeated switching between forward and reverse stress paths is likely to promote the development of internal back stress, which may contribute to the reduction in reverse yield strength [46,47]. With increasing loading cycles, the effects of residual internal stress and dislocation structures induced by prior plastic deformation are presumed to continuously accumulate, potentially leading to a gradual decrease in reverse yield stress and an enhanced Bauschinger effect. Once a certain number of cycles is reached, the reduction in reverse yield strength becomes progressively weaker, suggesting that the microstructural evolution may tend to saturate [48,49].

**Figure 30:**
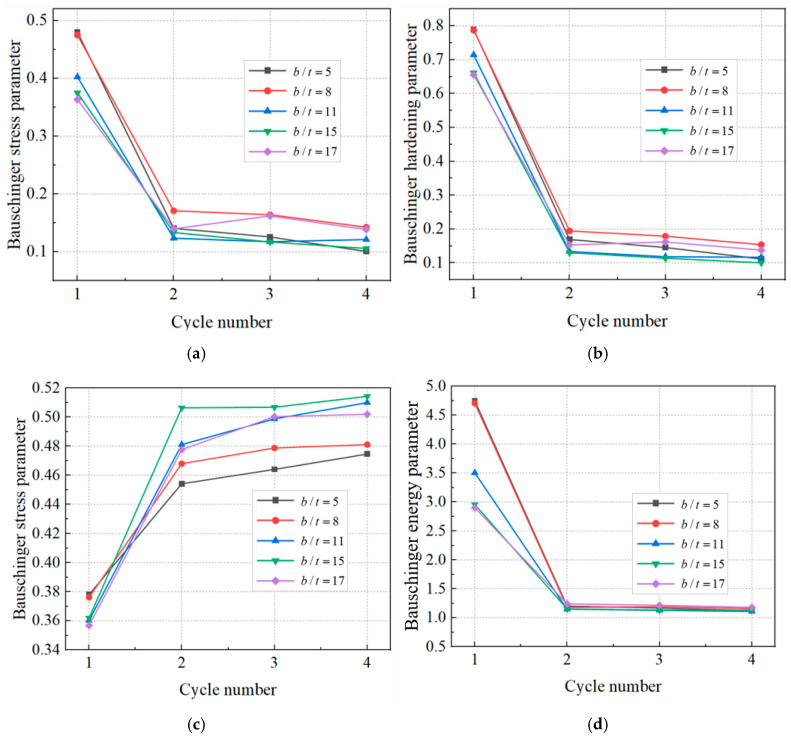
Comparison of Bauschinger-related parameters under different loading cycles. (**a**) Bauschinger ratio, B. (**b**) Bauschinger hardening parameter, BHP. (**c**) Bauschinger stress parameter, BSP. (**d**) Bauschinger energy parameter, BEP.

Figure 30 and Table 9 show the evolution of Bauschinger-related parameters under different loading cycles. With increasing cycles, the Bauschinger ratio B and Bauschinger hardening parameter BHP both decrease markedly. Their values are highest in the first cycle, drop rapidly after the second cycle, and then gradually stabilize. This indicates that the Bauschinger effect is pronounced during the initial reverse-loading stage, whereas reverse-yield softening and Bauschinger hardening weaken as cyclic plastic adaptation develops.

In contrast, the Bauschinger stress parameter BSP is relatively low in the first cycle, increases significantly in the second cycle, and then remains nearly stable, suggesting that the resistance to reverse deformation is rapidly established and reaches equilibrium after repeated loading–reverse loading in the early cycles. The BEP reaches its maximum in the first cycle, drops sharply in the second cycle, and changes only slightly thereafter. This indicates that the stress-path reversal is significant in the initial cycle, where the correction term plays a dominant role. With increasing cycles, the internal stress state and microstructural evolution may gradually stabilize, which likely explains why the correction effect weakens and tends to become constant [50].

**Figure 31:**
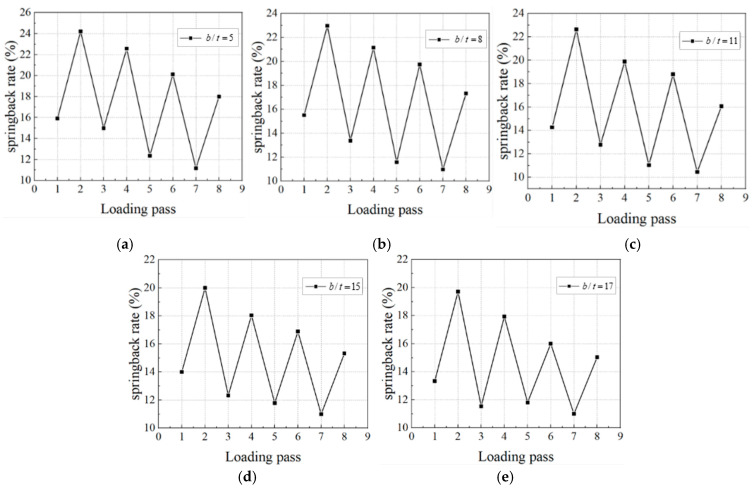
Variation in springback ratio with loading pass under different in-plane stress states. (**a**) b/t = 5. (**b**) b/t = 8. (**c**) b/t = 11. (**d**) b/t = 15. (**e**) b/t = 17.

Table 10 and Figure 31 show the variation in springback ratio with loading pass under different b/t conditions. For all conditions, the springback ratio in even-numbered passes is higher than that in the adjacent odd-numbered passes, while the overall value decreases with increasing loading pass. For example, at b/t = 5, the springback ratios decrease from 14.27% in the 1st pass and 24.22% in the 2nd pass to 11.17% in the 7th pass and 18.01% in the 8th pass, respectively. At b/t = 17, they decrease from 15.50% and 19.72% to 10.99% and 15.04%, respectively. These results indicate that cyclic loading history weakens the subsequent unloading springback capacity, while the reverse bending path consistently exhibits a higher springback ratio.

### 3.3. Effect of Target-Surface Pre-Strain on Bauschinger Effect and Springback

**Figure 32:**
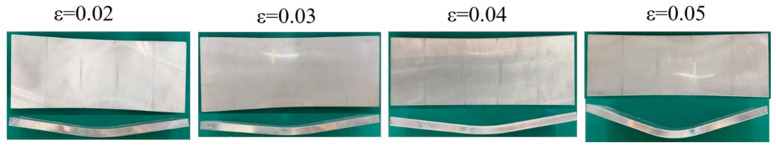
Experimental results of specimens under different strain conditions.

Pre-strain directly affects the plastic deformation of the target surface and its elastic recovery after unloading, and is therefore an important factor governing springback behavior. To isolate the effect of pre-strain, the thickness-to-width ratio is 8. Figure 32 shows the experimental results after one loading cycle under different target-surface strains.

**Figure 33:**
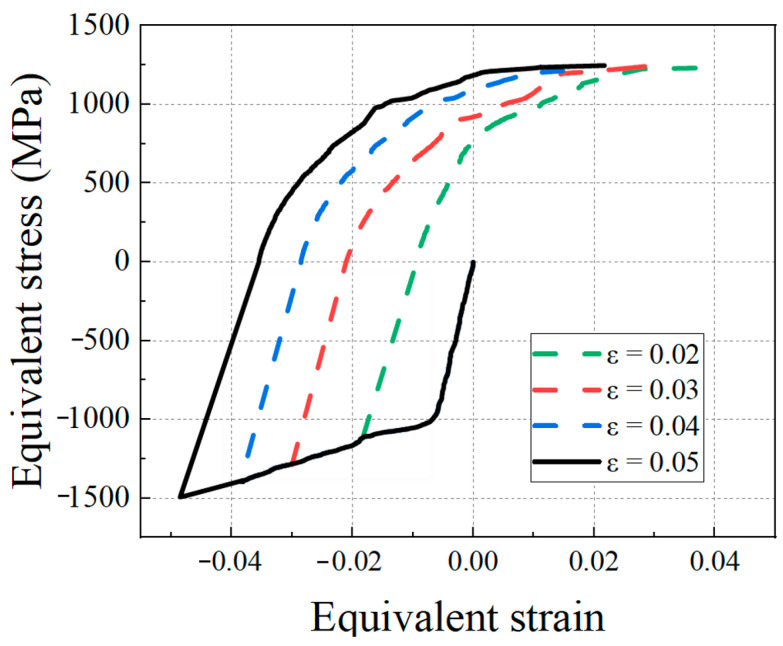
Equivalent stress–strain curves of the target surface under different pre-strains.

Figure 33 shows the equivalent stress–strain curves of the target surface under different pre-strains. During forward loading, the flow stress curves under different pre-strains almost overlap. During reverse loading, the yield stress decreases markedly, indicating a pronounced Bauschinger effect. After the prescribed strain is reached, the flow stress curves also tend to overlap, suggesting that the hardening response of Q890 steel during reverse bending gradually approaches saturation.

**Figure 34:**
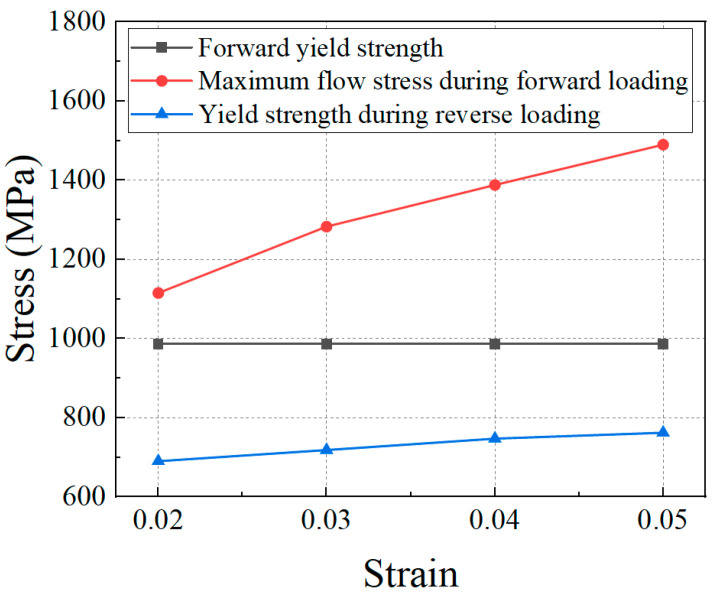
Target-surface characteristic stress parameters under different pre-strains.

To further quantify the response differences at different pre-strains, characteristic stress parameters were extracted, as listed in Table 11 and plotted in Figure 34. As the target-surface strain increased from 0.02 to 0.05, the maximum flow stress during forward loading increased significantly from 1099 MPa to 1490 MPa, while the reverse yield strength also increased from 703 MPa to 762 MPa. However, the reverse yield strength remained lower than the initial yield strength under all pre-strains, indicating that reverse yielding still occurred earlier than forward yielding and that a clear Bauschinger effect was present.

This may be attributed to the combined effects of work hardening and back-stress evolution during forward pre-deformation. A higher pre-strain is likely to promote dislocation multiplication and entanglement, thereby increasing the forward flow stress and raising the overall stress of the material. During subsequent reverse loading, the accumulated back stress and residual stress are presumed to still reduce the reverse yield strength relative to the initial yield strength. Nevertheless, the strengthening effect caused by increased plastic deformation appears to partially offset the reverse yield reduction, which may explain the gradual increase in the measured reverse yield strength with increasing pre-strain [51].

**Figure 35:**
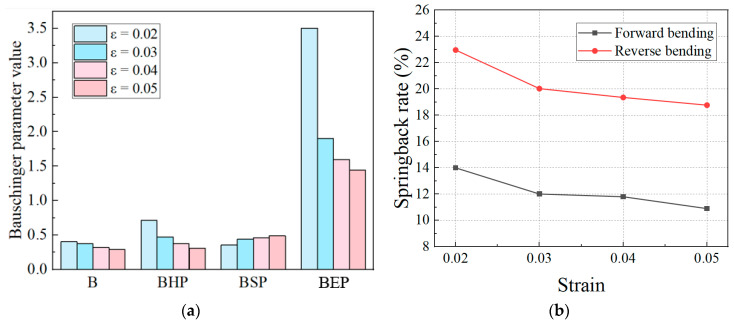
Variations in Bauschinger-related parameters and springback ratio under different target-surface pre-strains. (**a**) Bauschinger-related parameters. (**b**) Springback ratio versus target-surface strain.

The evolution of Bauschinger-related parameters and springback ratios under different pre-strains is summarized in Table 12 and Table 13 and Figure 35. The experimental results show that, under a constant stress ratio, both forward and reverse bending springback ratios decrease nonlinearly as the pre-strain increases from 0.02 to 0.05. The reverse bending springback ratio remains consistently higher than the forward bending springback ratio.

This can be attributed to the expansion of the plastic zone through the sheet thickness with increasing pre-strain, which reduces the proportion of recoverable elastic deformation after unloading. In addition, reverse bending is performed after prior forward pre-deformation. The residual stress, back stress, and Bauschinger effect developed during forward bending alter the stress distribution and yielding behavior during reverse loading, resulting in a higher reverse bending springback ratio.

As explicitly shown in Table 13, the magnitude of the decrease in the springback ratio was relatively large within the pre-strain range of 0.02 to 0.03. In this small-strain range, the transition from shallow to deeper plastic deformation is accompanied by rapid expansion of the plastic zone, making the springback ratio more sensitive to increasing deformation. As the pre-strain further increases, the material enters a deeper plastic deformation stage; consequently, the springback ratio continues to decrease, but the reduction becomes more gradual.

## 4. Comparison of Forming Accuracy of Large-Size High-Strength Steel Heavy Plates

**Figure 36:**
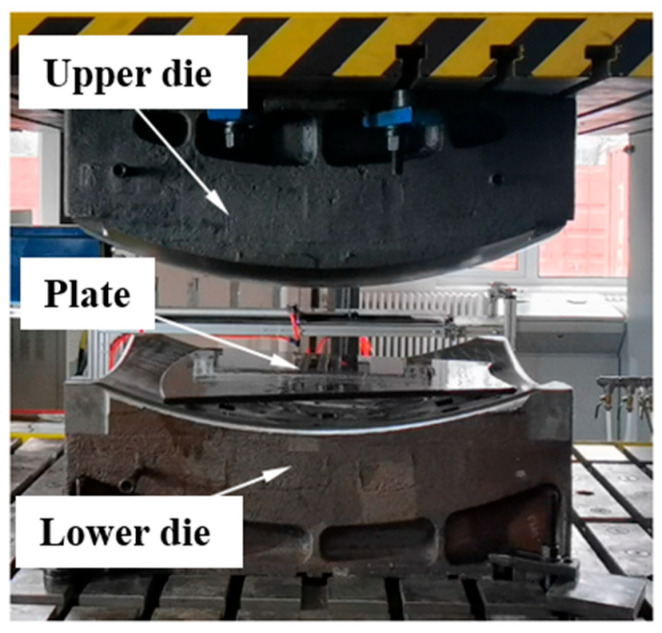
Q890 high-strength steel large thick plate whole press forming experimental mold physical drawing.

The actual die used for the integral press forming of the large-size Q890 high-strength steel heavy plate is shown in Figure 36. The shapes and dimensions of the upper and lower dies are consistent with those of the models in the finite element simulation. Both the upper and lower dies measure 1000 mm in length and 800 mm in width, featuring spherical surfaces with curvature radii of 1200 mm and 1216 mm, respectively. The dies are manufactured from ZG35Mn medium-carbon cast steel, the primary chemical composition of which is listed in Table 14. Prior to the experiment, the hardness of the dies was tested, yielding a result of approximately 210.6 HB. It has been verified that this hardness level is sufficient to meet the load-bearing requirements during the pressing deformation of the Q890 high-strength steel blank.

**Figure 37:**
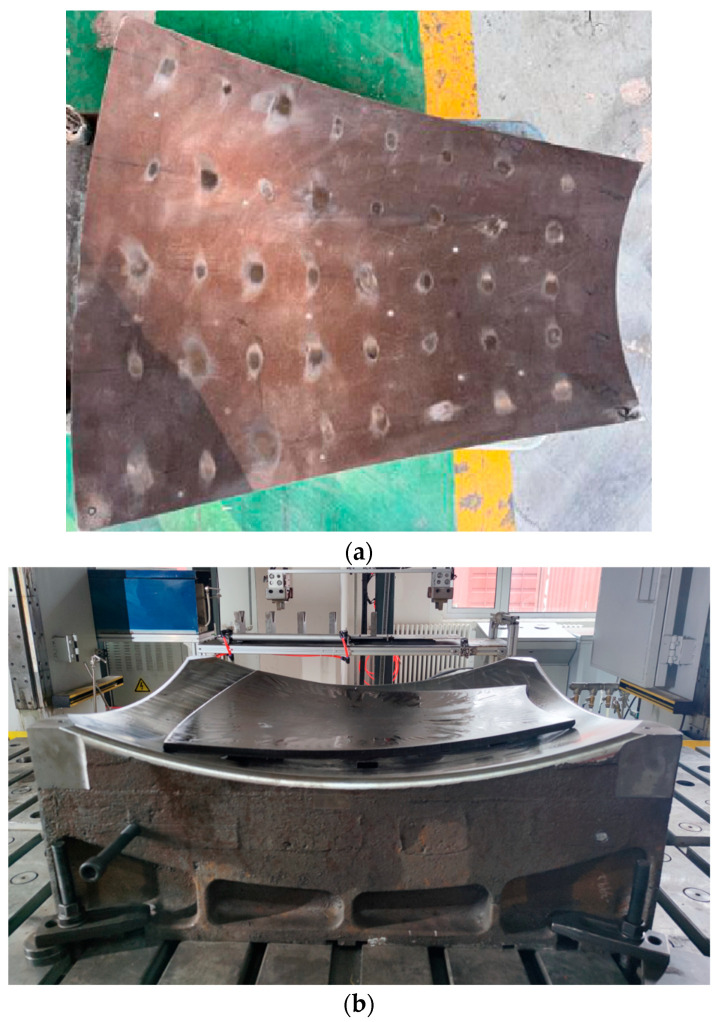
Formed part of the integral pressing test of large-size thick plates of Q890 high-strength steel. (**a**) Formed part of the press forming experiment of high-strength thick steel plate. (**b**) Springback behavior of the formed Q890 high-strength thick steel plate.

The formed component from the integral press-forming test is shown in Figure 37a. As observed from the gap between the formed component and the lower die (Figure 37b), the Q890 steel plate experienced pronounced springback upon die opening. To systematically evaluate the springback prediction accuracy, the Chaboche mixed hardening parameters calibrated using the proposed cyclic four-point bending method were compared with those determined from conventional cyclic shear tests. The actual inner surface of the formed component and the finite element results from both models were imported into a 3D reverse engineering verification software (geomagic qualify 2013). Subsequently, using the actual inner surface as the baseline, a 3D comparative analysis was conducted employing a best-fit alignment method.

**Figure 38:**
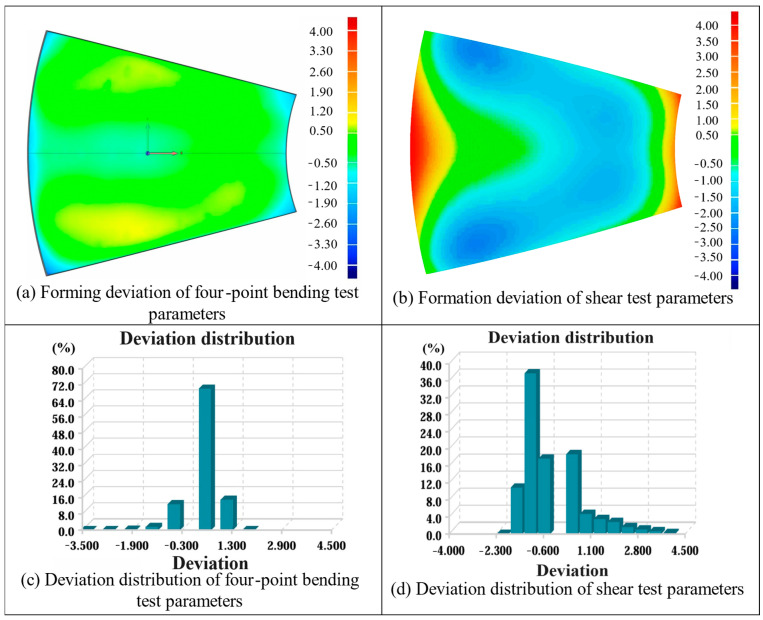
Comparison of FE and 3D comparison of forming results of large-size thick plate integral forming with different parameters.

Figure 38 presents the comparison between the finite element simulation results under different calibration strategies and the 3D scanning results of the actual formed component, along with a detailed statistical analysis of the corresponding deviations. Overall, while both predictions can reflect the global profile characteristics of the formed component, there is a significant difference in the concentration of deviations. As shown in the deviation distribution histograms, the simulated profile deviations using both sets of parameters are predominantly concentrated in the low-error interval near zero. Notably, the model utilizing the four-point bending test parameters exhibits the highest area proportion within the tight −0.50 to 0.50 mm interval, reaching approximately 70.0%. In stark contrast, the deviation distribution for the model using the shear test parameters is broader and more scattered, with its maximum proportion in the central primary peak interval reaching only about 38.0%.

This evidence directly substantiates that the constitutive model parameters calibrated based on the four-point bending path achieve a significantly higher degree of deviation concentration. Examining the extreme deviation intervals, the shear-calibrated model presents pronounced localized distributions of deep red and deep blue in the corner regions of the plate, retaining significant area proportions in the larger deviation intervals of less than −2.300 mm and greater than 2.800 mm. Conversely, the deviation contour of the bending-calibrated model displays a uniform green color overall. This demonstrates that when the Chaboche parameters are calibrated solely using the shear path, the model exhibits inherent limitations in predicting the profile recovery of the entire component after unloading. The proposed approach can more accurately predict the global springback profile of the large-scale curved heavy-plate component, thereby effectively guiding the actual forming process parameters.

## 5. Conclusions

This study proposed a cyclic four-point bending method for characterizing the Bauschinger effect of Q890 high-strength steel under in-plane biaxial cyclic loading. By adjusting the specimen width-to-thickness ratio, a series of biaxial stress ratios $\alpha_{t}$ were constructed on the target surface. Combined with DIC measurement and stress correction, equivalent stress–strain curves and Bauschinger-related parameters were obtained under different stress ratios, loading cycles, and target-surface pre-strains. The main conclusions are as follows.

(1) During cyclic four-point bending, the target surface was not subjected to an ideal uniaxial stress state, but exhibited pronounced biaxial stress characteristics. The stress ratios obtained in this study covered the dominant stress conditions encountered in forming large-scale double-curvature thick plates. Therefore, the stress ratio $\alpha_{t}$ is a key parameter for characterizing the in-plane biaxial stress state of the target surface and directly influences the mechanical response during subsequent forward loading, reverse loading, and unloading springback.

(2) The biaxial stress ratio $\alpha_{t}$, loading cycle, and pre-strain significantly affected the Bauschinger effect of Q890 high-strength steel during cyclic bending. As the Width-to-thickness ratio increased from 5 to 17, the equivalent yield stress decreased from 1098 MPa to 934 MPa, the forward maximum flow stress decreased from 1193 MPa to 1065 MPa, and the reverse yield strength decreased from 742 MPa to 685 MPa. Meanwhile, B, BHP, and BEP decreased from 0.479, 0.789, and 4.747 to 0.363, 0.655, and 2.900, respectively, revealing a strong stress-ratio dependence of the Bauschinger effect. When the pre-strain increased from 0.02 to 0.05, the forward maximum flow stress increased significantly from 1099 MPa to 1490 MPa, while the measured reverse yield stress also increased from 703 MPa to 762 MPa. However, the reverse yield stress remained lower than the initial yield stress, indicating that the Bauschinger effect was still evident.

(3) The springback behavior was jointly affected by the biaxial stress ratio $\alpha_{t}$, loading direction, cyclic history, and target-surface pre-strain. With increasing loading passes, the springback ratio showed a nonlinear decrease and gradually tended to stabilize. In addition, the springback ratio exhibited a distinct odd–even alternating characteristic, with even-numbered passes corresponding to reverse bending showing higher springback ratios than the adjacent odd-numbered passes corresponding to forward bending. When the pre-strain increased from 0.02 to 0.05, the springback ratios in both forward and reverse bending decreased overall, while the reverse-bending springback ratio remained consistently higher than that of forward bending. These findings demonstrate that in-plane biaxial stress-state effects must be considered when characterizing the Bauschinger effect and springback behavior of Q890 high-strength steel.

## Figures and Tables

**Table 1:** Chemical composition of Q890 high-strength steel (wt.%).

Element	C	Si	Mn	P	S	Cr	Ni	Mo	V
Content	0.11	0.35	0.40	0.015	0.010	0.45	4.40	0.32	0.12

**Table 2:** Mechanical properties of Q890 steel sheet.

Properties	0°	45°	90°
Young’s Modulus	217,000		
Poisson’s ratio	0.3		
Yield stress, σ_s_ (MPa)	1020	1015	1026
Ultimate tension strength, σ_uts_ (MPa)	1115	1106	1102
Lankford coefficient, r	0.97	0.98	1.01
Degree of planar anisotropy of plastic strain ratio, Δr	0.01		

**Table 3:** Chaboche mixed hardening model parameters for Q890 high-strength steel.

Material	*Q*	*b*′	*c*_1_	*γ*_1_	*c*_2_	*γ*_2_	*c*_3_	*γ*_3_
Q890	−190	0.85	158,865.8	1741	20,750.3	824.5	5778.8	33.8

**Table 4:** Specimen dimensions and corresponding width-to-thickness ratio.

Specimen No.	Specimen Size L × b × t (mm)	Width-to-Thickness Ratio b/t	Strain
S1	200 × 80 × 16	5	0.02–0.05
S2	200 × 80 × 10	8	0.02–0.05
S3	200 × 80 × 7.3	11	0.02–0.05
S4	200 × 80 × 5.4	15	0.02–0.05
S5	200 × 80 × 4.7	17	0.02–0.05

**Table 5:** Summary of uncertainty sources and estimated uncertainties of the main extracted quantities.

Uncertainty Item	Load Measurement	Displacement Measurement	Springback Angle Measurement	Corrected Stress	$\sigma_{Y}^{i}$	$\sigma_{r}^{i}$	BSP
**Estimated** **uncertainty**	±0.5%	$U_{rel}=0.3\%$	±0.2°	±2.0%	±2.0%	±2.0%	On the order of 10^−2^

**Table 6:** Characteristic stress parameters of the target surface under different stress states.

b/t	***σ***_Y_/MPa	***σ***_f_/MPa	***σ***_r_/MPa
5	1098	1193	742
8	1059	1151	718
11	986	1099	703
15	953	1086	693
17	934	1065	685

**Table 7:** Bauschinger-related parameters under different in-plane stress states.

Parameter	b/t = 5	b/t = 8	b/t = 11	b/t = 15	b/t = 17
B	0.479	0.474	0.402	0.375	0.363
BHP	0.789	0.787	0.714	0.661	0.655
BSP	0.378	0.376	0.360	0.361	0.356
BEP	4.747	4.706	3.504	2.954	2.900

**Table 8:** Characteristic stress parameters of the target surface under different loading cycles.

b/t	Number of Loading Cycles	$\sigma_{Y}^{i}$/MPa	$\sigma_{f}^{i}$/MPa	$\sigma_{r}^{i}$/MPa
5	1	1098	1193	742
	2	843	1354	739
	3	823	1364	731
	4	798	1380	725
8	1	1059	1151	718
	2	828	1329	707
	3	815	1343	700
	4	801	1351	701
11	1	986	1099	703
	2	772	1324	687
	3	751	1341	672
	4	739	1345	659
15	1	953	1086	693
	2	739	1321	652
	3	725	1316	649
	4	710	1322	642
17	1	934	1065	685
	2	733	1231	643
	3	724	1247	623
	4	706	1245	620

**Table 9:** Bauschinger-related parameters under different loading cycles.

Parameter	Loading Cycle	b/t = 5	b/t = 8	b/t = 11	b/t = 15	b/t = 17
B	1	0.479	0.474	0.402	0.375	0.363
B	2	0.140	0.171	0.123	0.133	0.139
B	3	0.125	0.164	0.117	0.117	0.162
B	4	0.100	0.142	0.121	0.105	0.139
BHP	1	0.789	0.787	0.714	0.661	0.655
BHP	2	0.169	0.194	0.133	0.130	0.153
BHP	3	0.145	0.178	0.118	0.113	0.162
BHP	4	0.111	0.153	0.116	0.100	0.138
BSP	1	0.378	0.376	0.360	0.361	0.357
BSP	2	0.454	0.468	0.481	0.506	0.478
BSP	3	0.464	0.478	0.498	0.506	0.500
BSP	4	0.474	0.481	0.510	0.514	0.502
BEP	1	4.747	4.706	3.504	2.954	2.901
BEP	2	1.203	1.180	1.149	1.153	1.245
BEP	3	1.170	1.193	1.128	1.133	1.219
BEP	4	1.125	1.159	1.111	1.132	1.182

**Table 10:** Variation in springback ratio with loading pass under different in-plane stress states.

b/t	1st Pass	2nd Pass	3rd Pass	4th Pass	5th Pass	6th Pass	7th Pass	8th Pass
5	14.27	24.22	12.78	22.56	11.80	20.11	11.17	18.01
8	14.00	22.97	12.34	21.14	11.58	19.73	10.97	17.32
11	13.33	22.64	11.54	19.89	11.03	18.79	10.45	16.06
15	15.91	20.00	13.36	17.94	11.79	16.89	11.00	15.32
17	15.50	19.72	14.98	18.04	12.37	16.01	10.99	15.04

**Table 11:** Characteristic stress parameters of the target surface under different pre-strains.

Strain	***σ***_Y_/MPa	***σ***_f_/MPa	***σ***_r_/MPa
0.02	986	1099	703
0.03	986	1283	718
0.04	986	1388	747
0.05	986	1490	762

**Table 12:** Bauschinger-related parameters under different target-surface pre-strains.

Strain	B	BHP	BSP	BEP
0.02	0.40256	0.71465	0.36033	3.50442
0.03	0.37326	0.47434	0.44037	1.90236
0.04	0.31995	0.37285	0.46182	1.59453
0.05	0.29396	0.30769	0.48859	1.44444

**Table 13:** Springback ratio under different target-surface pre-strains.

Strain	0.02	0.03	0.04	0.05
Forward bending	14.00	12.01	11.80	10.90
Reverse bending	22.97	20.03	19.36	18.77

**Table 14:** Chemical Composition of ZG35Mn.

Element	C	Si	Mn	P	S	Cr	Ni	Cu	Mo	V
Content	0.328	0.268	1.241	0.026	0.018	0.237	0.178	0.017	0.113	0.005

## Data Availability

The original contributions presented in this study are included in the article. Further inquiries can be directed to the corresponding authors.
